# Brain Hemorrhage Classification in CT Scan Images Using Minimalist Machine Learning

**DOI:** 10.3390/diagnostics11081449

**Published:** 2021-08-11

**Authors:** José-Luis Solorio-Ramírez, Magdalena Saldana-Perez, Miltiadis D. Lytras, Marco-Antonio Moreno-Ibarra, Cornelio Yáñez-Márquez

**Affiliations:** 1 Centro de Investigación en Computación, Instituto Politécnico Nacional, CDMX 07700, Mexico; soloriojoseluis@gmail.com; 2 Effat College of Engineering, Effat University, P.O. Box 34689, Jeddah 21478, Saudi Arabia

**Keywords:** eXplainable artificial intelligence, minimalist machine learning, image classification, machine learning

## Abstract

Over time, a myriad of applications have been generated for pattern classification algorithms. Several case studies include parametric classifiers such as the Multi-Layer Perceptron (MLP) classifier, which is one of the most widely used today. Others use non-parametric classifiers, Support Vector Machine (SVM), K-Nearest Neighbors (K-NN), Naïve Bayes (NB), Adaboost, and Random Forest (RF). However, there is still little work directed toward a new trend in Artificial Intelligence (AI), which is known as eXplainable Artificial Intelligence (X-AI). This new trend seeks to make Machine Learning (ML) algorithms increasingly simple and easy to understand for users. Therefore, following this new wave of knowledge, in this work, the authors develop a new pattern classification methodology, based on the implementation of the novel Minimalist Machine Learning (MML) paradigm and a higher relevance attribute selection algorithm, which we call *dMeans*. We examine and compare the performance of this methodology with MLP, NB, KNN, SVM, Adaboost, and RF classifiers to perform the task of classification of Computed Tomography (CT) brain images. These grayscale images have an area of 128 × 128 pixels, and there are two classes available in the dataset: CT without Hemorrhage and CT with Intra-Ventricular Hemorrhage (IVH), which were classified using the Leave-One-Out Cross-Validation method. Most of the models tested by Leave-One-Out Cross-Validation performed between 50% and 75% accuracy, while sensitivity and sensitivity ranged between 58% and 86%. The experiments performed using our methodology matched the best classifier observed with 86.50% accuracy, and they outperformed all state-of-the-art algorithms in specificity with 91.60%. This performance is achieved hand in hand with simple and practical methods, which go hand in hand with this trend of generating easily explainable algorithms.

## 1. Introduction

The prevention and early detection of diseases that affect health will always be a priority in the life of any person, given that poor health not only causes harm to each individual but also increases the costs of health institutions in each country. Information collected in 2019 by the World Health Organization (WHO), cardiovascular diseases, and diabetes mellitus are among the ten leading causes of death worldwide. Meanwhile, vehicle accidents are a cause of death in the vast majority of countries regardless of their level of economic income [1]. These conditions can lead to cerebrovascular diseases [2,3,4], which generally present in a condition known as intracranial hemorrhage or cerebral hemorrhage. Unfortunately, for those who suffer from this condition, the warning signs or symptoms with a slight degree of hemorrhage are practically imperceptible, which in many occasions generates the aggravation of the disease.

Normally, the initial evaluation of these kinds of organic anomalies by medical specialists is usually performed by means of diagnostic imaging techniques, commonly Magnetic Resonance Imaging (MRI) and Computed Tomography (CT). These two modalities are widely used to produce images with great quality and detail of brain structures, because through the use of contrasts applied to patients, it is possible to differentiate the elements that make up the anatomical structure of human beings. CT is particularly useful in identifying patients in whom brain damage or injury has occurred, who regularly demonstrate some degree of cerebral hemorrhage, as well as ventricular abnormalities. MRI has the characteristic of better and more sensitive imaging of different areas of the brain, such as white matter, gray matter, corpus callosum, deeper areas, ventricular areas, and the brainstem; nowadays, more patients undergo MR imaging after having suffered an injury [5]. However, the preparation for acquiring these images is much more complex than CT, so in acute phases, CT is the modality of choice when brain injury is suggested [6]. Unfortunately, however, these imaging techniques are sometimes not sufficient to determine the suspicion of a brain anomaly, and it is essential to obtain more information about its characteristics, requiring the invasion of the tissue by means of a craniotomy, so that the specialist can make a detailed diagnosis [7].

CT images for the diagnosis of cerebral hemorrhages are the most widely used for the detection of this condition due to their high quality. These cerebral hemorrhages have various causes of occurrence, such as cranioencephalic trauma, hypertension, or intracranial tumors. In general, they are usually divided into five types of hemorrhages, depending mainly on the area where they occur [8].

- Epidural Hemorrhage (HED);
- Subdural Hemorrhage (SDH);
- Subarachnoid Hemorrhage (SAH);
- Intracerebral Hemorrhage (ICH); and
- Intraventricular Hemorrhage (IVH).

By implementing images captured by increasingly sophisticated means, such as MRI and CT, together with engineering technologies and Artificial Intelligence (AI), it has been possible to generate a large number of computer vision applications, where intelligent computation can be integrated with these images to design and develop complex diagnostic systems and thus help physicians to increase the diagnostic accuracy of early detection of various diseases. Techniques such as Artificial Neural Networks (ANN), Support Vector Machines (SVM), and Deep Learning algorithms have been applied to visual pattern recognition, as in the case of brain hemorrhages [9,10].

The main disadvantage of Deep Learning algorithms is that they work ideally with a very large number of training instances or examples, and in particular examples, the deeper a network is, the more accurate it will be [11]. However, this makes it practically impossible to know how these algorithms converge to a solution, which turns them into a Black Box.

Within the context of Artificial Neural Networks, this is where most of the efforts of the scientific community have been focused, specifically for the classification of images by Convolutional Neural Networks (CNN). This is due to the marvelous performance they have maintained for the aforementioned task, and the large number of models that currently exist; compared to methodologies based on the study of the characteristics of an image, such as the radionomic study of an image, the performance of algorithms based on Machine Learning [12] is far superior. However, a very important concept in data analysis is neglected: simplicity.

The excellent performance of these Deep Learning algorithms is not really up for debate, but their opacity and comprehensibility leave a virgin space within the area of Artificial Intelligence. In view of this, and with the efforts of several organizations worldwide, a new current was generated, which was called eXplainable Artificial Intelligence (XAI).

XAI was created with the primary intention of generating AI systems that provide accessible explanations of the way in which algorithms make a given decision, thus creating systems that are highly reliable and, in turn, more transparent compared to the usual AI systems.

Naturally, an important question arises: when can an algorithm be considered explainable? In response to this question, extensive discussions have led to the establishment of five characteristics that these models must satisfy [11].

- Interpretability means providing explanations to end users for a particular decision or process.
- Transparency quantitatively measures the ease or accessibility of a model or algorithm.
- Explainability allows an explanation to be given of how a particular model has taken a particular solution.
- Contestability means that any XAI user can affirm or reject a decision taken.
- Justifiability indicates an understanding of the case to support a particular outcome.

This need to make Machine Learning models more transparent and explainable has inspired us to carry out this work, as we naturally think that the task of image classification is complex and, above all, extensive in its development. This motivated us to develop a methodology that makes use of two of the most relevant measures within an image [12], and the automation of the classification task by means of an unconventional paradigm, which is known as Minimalist Machine Learning. This timely algorithm allows us to go beyond simplicity, of course without leaving aside the excellent results that our main proposal allows us to achieve, compared to algorithms that currently obtain very good results. Among these algorithms are mainly CNNs, whose creation is focused on the classification of images, mainly in the detection of diseases [12,13], as the great effectiveness and versatility shown has been evident [13]. However, consequently, it has sacrificed simplicity in its learning and understanding, becoming practically a black box, of which what goes in and what comes out is known to perfection, but unfortunately, what happens inside is difficult to understand.

In the same way, it is important to mention that in the field of Deep Learning, efforts are being made to take these models based on Artificial Neural Networks to the path of explainability, mainly in areas of major relevance such as health [14].

A clear example of this is the work done by Wang et al. In this work, the efficient detection of small brain hemorrhages is achieved by analyzing structural abnormalities found on MR images. The authors succeeded in developing a practical model with very good performance. They achieve this with a nine-layer [15] CNN model, obtaining values above 97% in four different performance measures. Subsequently, Doke et al. carried out a work dedicated to the classification of micro-bleeds at the brain level using Convolutional Neural Networks and Bayesian optimization [16]. This excellent work is close to the fundamental concept of the development of this paper, as a simple CNN model is developed with only five layers, basic operators, and filters, which are developed in a very efficient way. These examples show the panorama of the increasingly extensive use of machine learning models in tasks such as anomaly detection or brain diseases, but they still leave a wide field in the generation of simpler and more practical algorithms, without neglecting efficiency, which is ultimately the main purpose of any of the classification models.

In the MML paradigm, one of the most applied tasks in the area of Pattern Recognition (PR) is performed: classification. An important characteristic of this paradigm is the ability to represent the results and data graphically in a Cartesian plane. In order to achieve this graphical representation, statistical measures are used to identify the classes, as reflected in the article Toward the Bleaching of the Black Boxes: Minimalist Machine Learning [17], where the author obtains the statistical properties of the data: Arithmetic Mean and Standard Deviation.

In the context of information obtained from images, it is possible to define a set of characteristics that can support the determination of the behavior of an image, presenting a series of elements that can distinguish a delimited set of instances, through more complex attributes not related to mathematical operations, with an observable behavior in all images. These quantifiable observations are areas, solidity, perimeters, contrast, energy, entropy, and orientation, to mention some of the most outstanding [18].

By means of these attributes present in the images, each instance has the possibility of having different values for each behavioral attribute of the image, creating the possibility of being identifiable, identified, or classified by means of supervised learning algorithms.

Supervised learning consists of training a set through operations or calculations, and this process is known as the training or learning phase, which allows obtaining an optimal hypothesis, which achieves the best generalization and approximation according to the training data; on the other hand, there is the test set, which consists of instances that will be subjected to a classification phase and compared with the hypothesis resulting from the training phase, in order to assign a label to this test element that was completely unknown during the learning phase [19].

Based on the above, a classifier can be defined as a system that categorizes instances contained in a finite set of classes, which is fed by an array of attributes and whose output is a label associated to the input in question, which is assigned according to a maximum similarity function [20]. A Pattern Recognition (PR) model must have the ability to decide which label to assign to a test pattern that has never before been observed by the system, given a finite set of data provided at the input of the classification model. In this classification model, the following tasks are performed [21]: characterization and obtaining samples (always with the support and assistance of an expert in the field), grouping of the samples into their respective classes, according to the group formation criteria, training of the algorithm, and finally the validation of the model using validation methods, which allow evaluating the performance of the algorithm with test patterns whose class is unknown.

The main contribution of this paper is to present a simple and practical proposal for image classification, whose application in medical images simplifies the classification task and allows visualizing in a two-dimensional plane the behavior of a dataset, without leaving aside the very good performance achieved by this methodology compared to the state-of-the-art classifier algorithms.

The remainder of the paper consists of the following sections. Section 2 includes some of the related methods that will support the work presented. Section 2 is made up of four subsections, where some materials and methods are included. Section 2.1 includes brief information about various image enhancement techniques. Section 2.2 describes the process followed for image segmentation based on the implementation of morphological operators. Section 2.3 presents some theoretical advances that have allowed the generation of an efficient classification algorithm based on the MML paradigm. Moreover, the content of Section 2.4 serves as a solid base on how the learning and classification process of a new classification algorithm of this new MML paradigm is carried out. Section 3 presents the main proposed methodology of the work. Section 3.1 deals with CT image enhancement and image segmentation. Section 3.2 includes the methodology of applying the classification algorithm of the Minimalistic Machine Learning paradigm. Finally, Section 3.3 describes by means of real examples with two numerical datasets the way this new paradigm works. Section 4 presents the results. Section 4.1 describes the image dataset that is evaluated under the MML paradigm. In Section 4.2, we show in broad strokes the strong classifiers found in the state of the art, under which our results are compared. Section 4.3 shows the performance achieved, performance measures, and a brief analysis of the different results achieved. Section 5 contains conclusions and future work. Finally, references are included.

## 2. Materials and Methods

Several methodologies have been proposed to address the problem of CT image classification for the detection of intracerebral hemorrhages. For the development of this work, we use a series of image enhancement treatments; a novel algorithm that allows us to select the most relevant attributes that make up a dataset; and the main proposal of this work, which consists of the implementation of a classification algorithm of the MML paradigm. Section 2.1 describes in more detail the image equalization and image filtering techniques. Section 2.2 describes the application of erosion and dilation operators on images. Section 2.3 discusses in detail the operation of the *dMeans* data transformation technique. In addition, the fundamental methodology, the MML algorithm is described in Section 2.4.

### 2.1. Image Processing

#### 2.1.1. Contrast Limited Adaptive Histogram Equalization (CLAHE)

Contrast enhancement in an image by Contrast Limited Adaptive Histogram Equalization (CLAHE) is a widely used technique for medical image processing [22]. Particularly, CLAHE increases the gray intensity levels in those areas whose values are almost constant; this is relevant because it includes areas with the presence of noise, causing the problem of noise amplification to be reduced. In paper [23], Muniyappan, Allirani, and Saraswathi used CLAHE to enhance medical images; under this method, the maximum values that exceed a certain threshold are trimmed and then redistributed in the rest of the image. This redistribution parameter, commonly applied to histograms with bell-shaped behavior, is known as the Rayleigh distribution parameter.

The effect of the CLAHE technique is primarily that it selectively enhances and keeps the edges of each area sharper, mainly in those areas where there is not a large difference in the surrounding gray intensity [24]. This enhancement produces an increase in contrast levels, generating better quality images, exposing areas of possible interest with better detail, and making it an excellent solution for improving the quality of a CT image [25]. Although CLAHE also reduces the level of noise in the images, it is essential to apply a suitable filter to ensure acceptable quality for the main purpose.

#### 2.1.2. Mean Filter

Special image filtering consists of a set of products and sums performed on an image, using a mask, which is also known as a kernel [26]. The purpose of this kernel is to perform operations on a finite number of neighboring pixels. Defining the coordinates of an arbitrary point as (x,y), the result of the filtering is set as g(x,y).

(1) g(x,y)=w(−1,−1)f(x−1,y−1)+w(−1,0)f(x−1,y)+⋯+w(0,0)f(x,y)+⋯+w(1,1)f(x+1,y+1)

The default kernel traverses the entire image, so that w(0,0) is located in the pixel will get the result of the filtering. In general, the path and result will be determined by an image *M* and a kernel of dimension *m*.

(2) $$g\left(x,y\right)=\overset{a}{\underset{s=-a}{\sum }}\overset{b}{\underset{s=-b}{\sum }}w\left(s,t\right)\cdot f\left(x+s,y+t\right)$$

**Figure 1:**
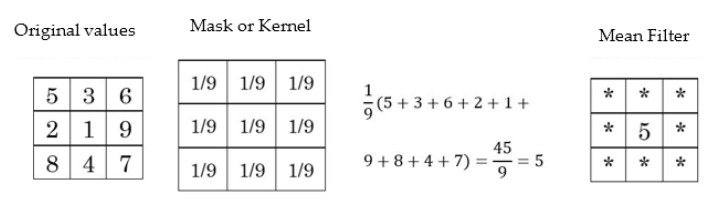
The pixel is replaced by the mean value of the nine nearest neighbor values.

Particularly, the mean filter, which contemplates that the kernel goes through the whole image, and the result of the product of each element by a pixel of the corresponding image are calculated to obtain the average of these and to be placed in the position w(0,0) corresponding to the image. Figure 1 shows a simple example of how the mean filter is applied to a given pixel.

The use of the mean filter is optimal for reducing random noise, which is better known as salt and pepper noise [27]: a type of noise found in images from digital devices, such as CT image acquisition [28]. It is also a fast filter, as it simply removes the noise without seeking to separate frequencies, as is the case with other types of filters. Moreover, its versatility allows it to be iterated a number of times in an efficient way, and with results very close to more sophisticated filters [29]. This makes the choice of this filter in CT images an excellent option for a simple and fast implementation. By using this technique, we can guarantee that the noise present in each image appears and an optimal image quality is maintained, which is useful for the segmentation process of areas of interest.

### 2.2. Morphological Operations

The morphological operations are based on Set Theory operations [26]. The images are treated as subsets of the space ${\mathbb{Z}}^{2}$. The purpose of these operations is to simplify and preserve the main shape characteristics of the objects.

#### 2.2.1. Erosion

Erosion works with two substantial elements: the first is a finite segment of the original grayscale image, i.e., image I containing a defined set A, and the second is a (usually smaller) set of coordinate points, called structuring element B [30,31]. They are mathematically defined by the following expression:

(3) $$A⊖B=\{z\;|\;{\left(B\right)}_{z}\subseteq A\}.$$

**Figure 2:**
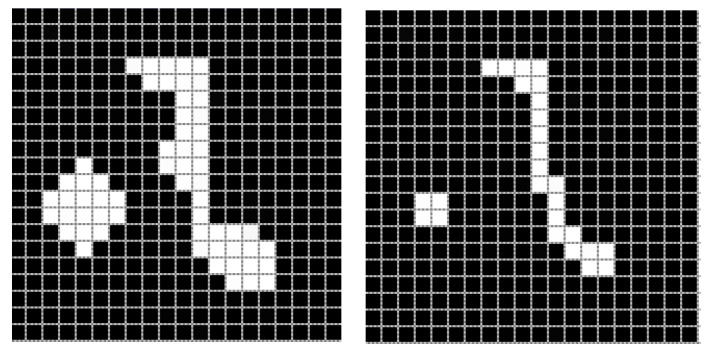
Erosion effect on an example image, applying a structuring element of dimension 3×3.

To calculate the erosion of a binary image by its structuring element, we use each pixel of the set A. For each object pixel, the structuring element is superimposed so that the origin of the structuring element coincides with the coordinates of the input pixel. If each pixel of the structuring element corresponds to that of the object, then the input value retains its value; otherwise, the object pixel takes the value of the image background (in binary images, the value zero corresponds). Figure 2 shows the effect of applying erosion to a binary image.

#### 2.2.2. Dilation

The binary dilation is considered the dual operation of erosion, in the same way it consists of a fundamental set, which is the image I, and from this is derived a subset A, to which the dilation operation will be applied with respect to a structuring element [32]. A and B are part of a set ${\mathbb{Z}}^{2}$ [30]. The mathematical expression that describes it is the following:

(4) $$A\;⨁\;B=\{z\;|\;{\left(\hat{B}\right)}_{z}\;{⋂}^{\;}\;A\;\neq \emptyset \}.$$

**Figure 3:**
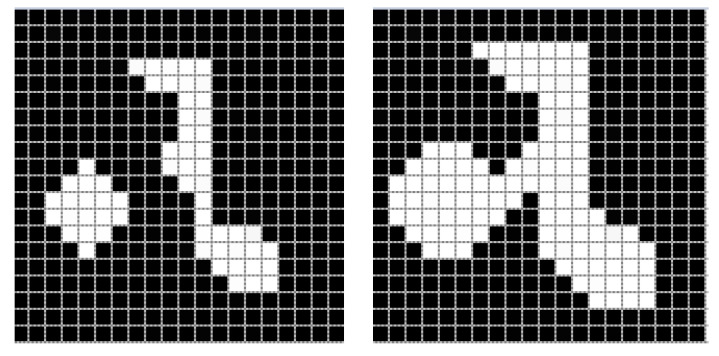
Dilation effect on an example image, applying a structuring element of dimension 3×3.

The effect of this operator is observed with the gradual enlargement of the boundaries of the defined regions of the objects. In this way, the pixels of the object increase in area while the existing spaces or gaps are reduced. The degree of dilation depends directly on the dimensions of the structuring element. Figure 3 shows the effect of a structuring element on a test image.

### 2.3. dMeans

The *dMeans* transformation is an algorithm that consists of performing a set of operations that favor the separability of the classes that make up the dataset; the operations implemented for this transformation are standard deviation and arithmetic mean. This transformation is only applied to the training set.

To apply this methodology, an assumption is made about the dataset with numerical features in its entirety and without the presence of missing data. It is established that it consists of two mutually exclusive classes. Applying the validation method, the dataset is divided into the sets $T_{r}$ (training or learning) and $T_{e}$ (testing or classification).

The *dMeans* algorithm takes as input the set $T_{r}$. From $T_{r}$, it detects the most relevant features of the dataset, generating what we will call Relevance Permutation (RP). In Algorithm 1, the operation of the *dMeans* algorithm is shown.
**Algorithm 1:***dMeans* algorithm.**Input:**$T_{r}$ training set, with two mutually exclusive classes $C=\left\{C_{1},\;C_{2}\right\}$. The attributes are formed by a set of attributes $A=\left\{A_{1},\;A_{2},\;A_{3},\;\ldots ,\;A_{n}\right\}$
**1.** For each attribute $A_{i}$
**(a)** Compute the mean $M_{1i}$ of the values of the attribute $A_{i}$ of all the patterns belonging to class $C_{1}$**(b)** Compute the mean $M_{2i}$ of the values of the attribute $A_{i}$ of all the patterns belonging to class $C_{2}$**(c)** Compute the difference $M_{1i}-M_{2i}$ and store that value in $d_{i}$
**2.** Create the RP sorting the $d_{i}$ values from high to low.
**Output:** n positions, which are sorted in descending order, by its relevance.

### 2.4. Minimalist Machine Learning

In order to apply this novel algorithm based on the Minimalist Machine Learning (MML) paradigm, it is essential to establish that each of the images that make up the dataset go through a flattening process, forming a two-dimensional array of size m×n where m is the number of patterns and n is the number of attributes of each pattern.

**Figure 4:**
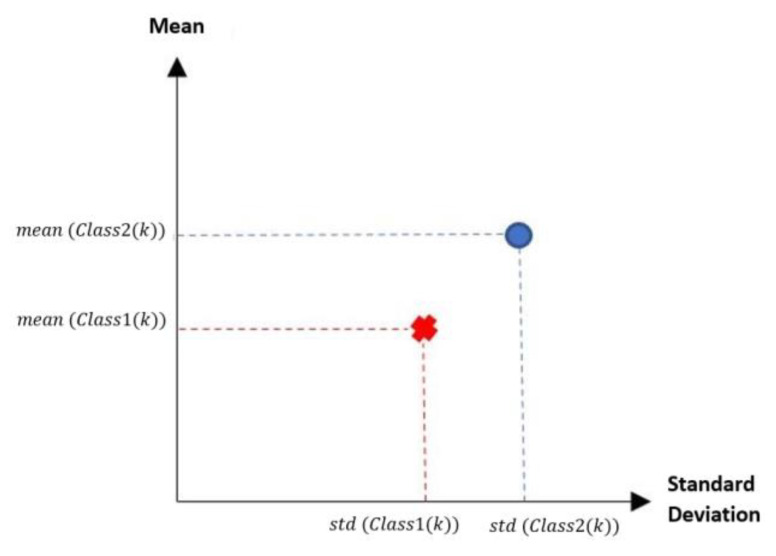
Location of a transformed pattern of each class on the Cartesian plane.

According to the MML paradigm, each of the patterns will go from having n attributes to only two attributes. These two attributes are real numbers, which are calculated by the standard deviation and mean of each of the patterns [17], thus becoming a dataset of size m×2. The first attribute corresponds to the calculated standard deviation value, and the second attribute corresponds to the calculated mean value. By performing these two calculations, it is possible to visualize in the Cartesian plane the behavior of each of the patterns. The x-axis corresponds to the standard deviation value, while the y-axis is conditioned to the mean. Figure 4 shows an example of how a pattern of each class can be visualized in the Cartesian plane.

**Figure 5:**
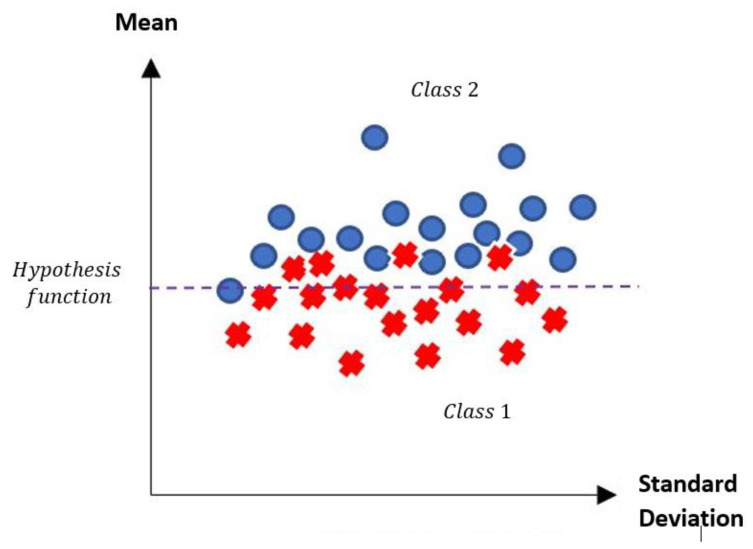
Exemplification of the location of each of the patterns on the Cartesian plane.

Figure 5 is a representation of what we seek to observe with any dataset that meets the criterion of being made up of numerical values. Next, the two phases that make up the MML paradigm will be described. These two phases are the training phase and testing phase.

In the field of pattern recognition, specifically under the supervised learning paradigm, an intelligent pattern classifier essentially consists of two phases: the training or learning phase, and the testing or classification phase [19]. To simplify the identification of the training and test sets derived from the original dataset, $T_{r}$ is referred to as the training set and $T_{e}$ is referred to as the test set.

## 3. Proposed Methodology

In this section, the methodology implemented in this work is presented. In Section 3.1, the proposed techniques for image enhancement are briefly described. Section 3.2 describes the MML paradigm including the *dMeans* algorithm and its decision boundary. Finally, Section 3.3. will show a series of illustrative examples that will help to better understand the learning and testing phases of the developed model. 

### 3.1. Image Enhancement

The improvement of the images is one of the most relevant stages, since a deficient preprocessing would lead to a drastic decrease in the quality of any classifier, no matter how good the results may be, so the techniques used to improve the quality of the images and, above all, to highlight those characteristics that will be most useful for the classifier will be shown below. The proposal made for image processing aims to improve the quality of the images, so that the greatest number of attributes of interest can be extracted, discarding those that are not useful for the further development of the work [33]. In Section 3.1.1, Section 3.1.2, Section 3.1.3 and Section 3.1.4, the procedures applied to the images will be detailed.

#### 3.1.1. Contrast Limited Adaptive Histogram Equalization (CLAHE)

As an alternative to using histogram equalization (usually by applying the *histeq* function), by coding in MATLAB, we use an enhancement known as Contrast Limited Adaptive Histogram Equalization using the *adapthisteq* function [34]. While *histeq* works on the entire image, *adapthisteq* operates on small regions of the image, which are called mosaics. The contrast of each mosaic is enhanced so that the histogram of the output region roughly matches a specified histogram. After equalization, *adapthisteq* combines neighboring mosaics by bilinear interpolation to remove artificially induced boundaries.

**Figure 6:**
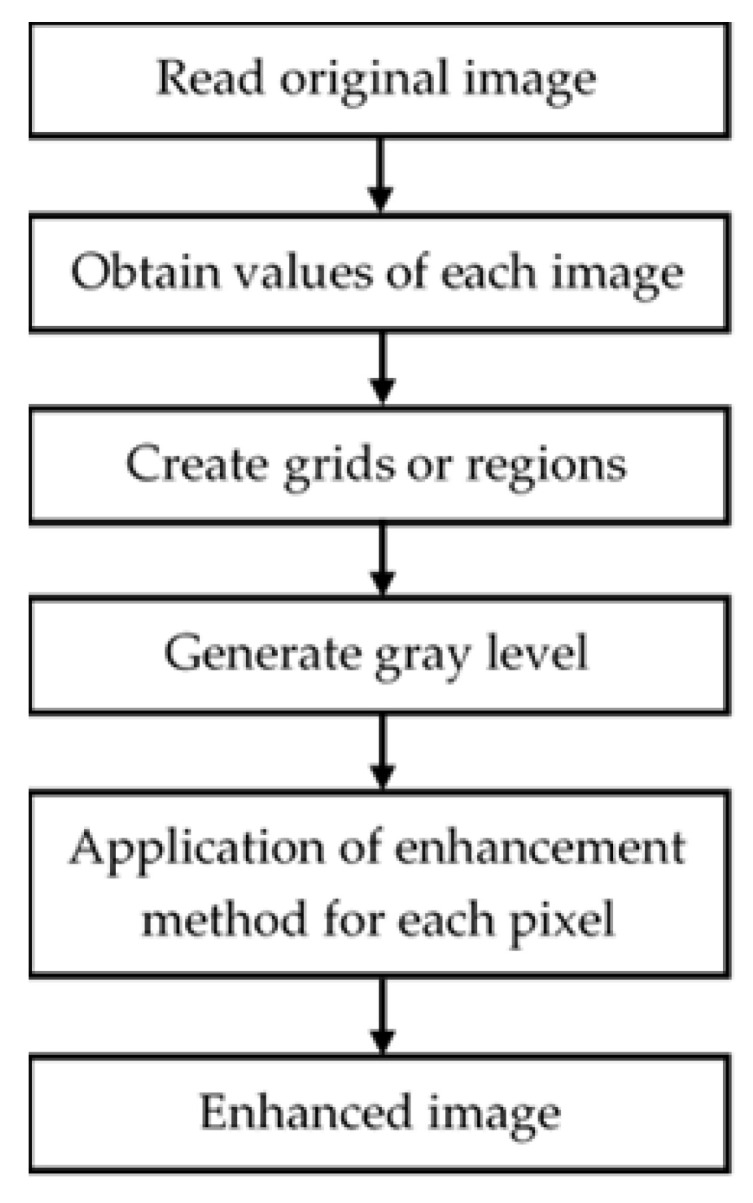
Process to apply CLAHE algorithm.

To avoid the amplification of any noise that may be present in the image, you can use the optional *adapthisteq* parameters to limit the contrast, especially in homogeneous areas. Figure 6 illustrates the operation of the CLAHE algorithm.

In this paper, we used this method to create a smoothing effect on the histogram of each image. This effect produces an improvement in contrast levels, generating better quality images.

#### 3.1.2. Mean Filter

**Figure 7:**
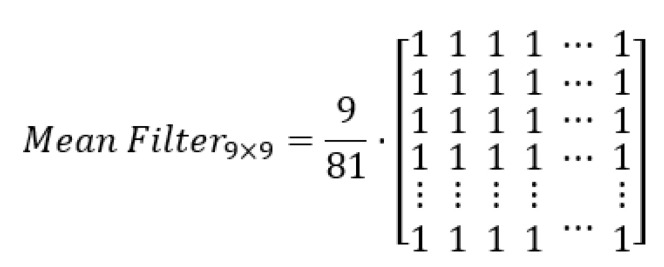
9 × 9 dimension median filter used for noise filtering in each image.

Once the bone tissue has been extracted from the image, additive noise filtering is performed using a 9 × 9 pixels median filter (Figure 7), thus smoothing the image and avoiding Gaussian noise (noise generated by electronic systems and sensors) [35]. Although this filter could be applied from the reading of the image, it is intended to eliminate those sections of bone tissue left over after its removal.

The dimensions of the filter are justified by the dimensions of the image, because due to its significant size, the excess segments or noise will be proportional to its dimensions. It was chosen because it is able to fulfill two functions: first, to smooth the image and second, to act as a low-pass filter that eliminates the additive noise presented.

#### 3.1.3. Removing the Skull

**Figure 8:**
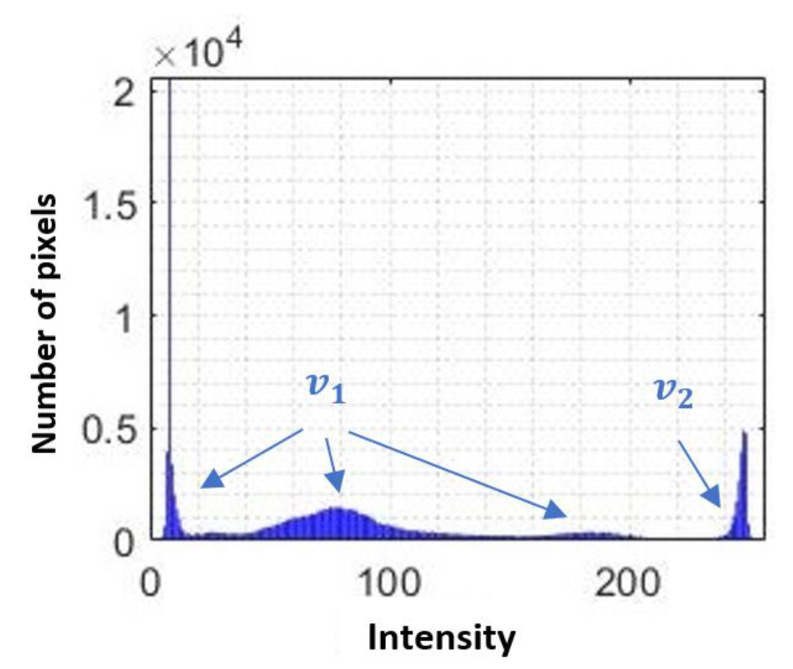
Original histogram of a class IVH image.

Within a brain CT image, different types of tissue can be captured. These tissues can be differentiated by the solidity that characterizes them, such as the encephalic mass and the bone structure or skull. As can be seen in Figure 8, 4 peaks are identified in the histogram of an image showing Intra-Ventricular Hemorrhage.

The first 3 peaks that make up the group $v_{1}$ are the soft tissues that make up the brain mass, and in the group $v_{2}$, the areas of greater intensity are identified: intensities that characterize the bone tissue [33].

Knowing the characteristics of the image, it is feasible to apply intensity-based segmentation. This technique consists of assigning binary values to a grayscale image, this assignment corresponds to a specific value, known as threshold value (α), which serves as a limit. When a grayscale value is greater or less than the reference value, it is assigned a value determined by the equation g(x,y) in order to eliminate the bone tissue and preserve the areas of interest. This segmentation is also known as global segmentation, and the expression that defines it is given by [36]:

(5) $$g\left(x,y\right)=\{\begin{array}{c} 0\;\;\;\;\;\;\;\;\;\;\;\;\;\;\;\;\;\;\;\;\;\;if\;\;f\left(x,y\right)>\alpha \\ f\left(x,y\right)\;\;\;\;\;\;\;\;\;\;\;if\;\;f\left(x,y\right)\leq \alpha \end{array}$$

where g(x,y) is the image resulting from the application of intensity-based segmentation, f(x,y) is the source image, and α is the threshold value that will determine the assignment of a binary value. The generalized α value was calculated as the average of the intensity value of a reduced set of images. This kind of segmentation will make it possible to extract relevant information and discriminate that which is not useful.

**Figure 9:**
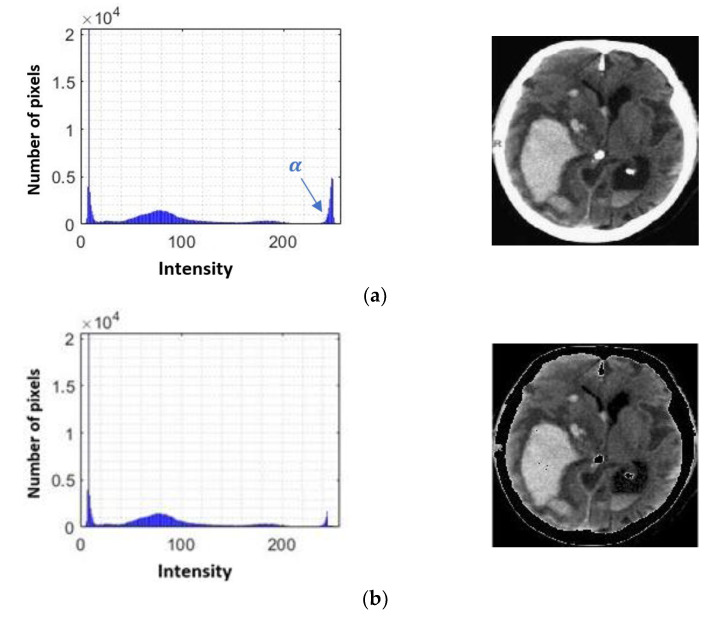
Observation of the histogram and segmentation result with the proposed α value. (**a**) Histogram of original image; (**b**) histogram of the image without bone tissue.

Figure 9 shows an example of the representation of the α level over the histogram for a brain CT image without hemorrhage and with the presence of hemorrhage and the effect caused by image segmentation.

#### 3.1.4. Segmentation of Possible Hemorrhages

The implementation of a global thresholding can be extended to a multiple definition, where a well-defined interval of gray levels can take a single value that allows identifying or intensifying zones of greater interest. For this purpose, two limits are generated: a lower limit $\alpha_{1}$ and an upper limit $\alpha_{2}$. This implies that elements between the interval $\alpha_{1}\geq f\left(x,y\right)\geq \alpha_{2}$ get a certain value that highlights them [36]. The following expression describes how the values of each image element are reassigned.

(6) $$g\left(x,y\right)=\{\begin{array}{c} 0\;\;\;\;\;\;\;\;\;\;\;\;\;\;\;\;\;\;\;\;\;\;if\;f\left(x,y\right)<\alpha_{1}\\ 255\;\;\;\;\;\;\;\;\;\;\;if\;\alpha_{1}\leq f\left(x,y\right)\leq \alpha_{2}\\ 0\;\;\;\;\;\;\;\;\;\;\;\;\;\;\;\;\;\;\;\;\;\;if\;f\left(x,y\right)>\alpha_{2} \end{array}$$

where g(x,y) is the image resulting from the application of intensity-based segmentation; f(x,y) is the source image; $\alpha_{1}$ is the lower segmentation limit that will determine the assignment of a given value; and $\alpha_{2}$ is the upper segmentation limit. The generalized $\alpha_{1}$ and $\alpha_{2}$ values were calculated by obtaining the average of the intensity values of a reduced group of images. This kind of segmentation will allow us to extract areas where hemorrhages exist more frequently, based on the level of gray intensity, in addition to discriminating those areas that are not useful.

**Figure 10:**
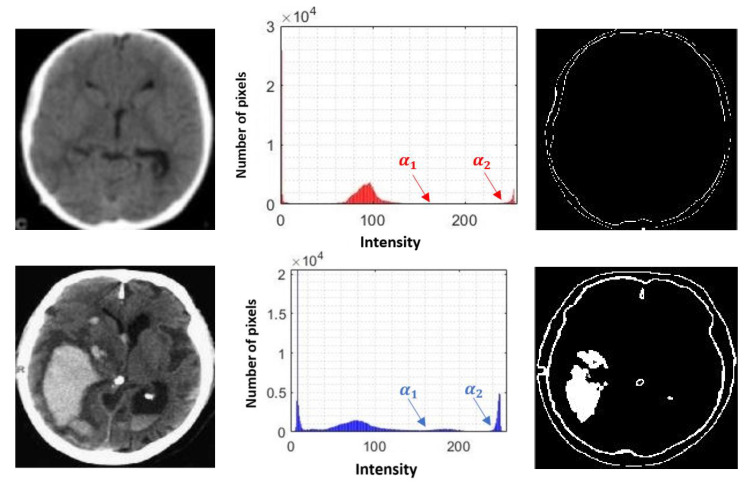
Observation of the histogram and segmentation result with the proposed α value.

Figure 10 shows an example of the representation of the $\alpha_{1}$ and $\alpha_{2}$ levels on the histogram for brain CT images without hemorrhage and with the presence of hemorrhage, which were derived from the image segmentation.

In the field of pattern recognition, specifically under the supervised learning paradigm, an intelligent pattern classifier consists essentially of two phases: training or learning phase, and testing or classification phase. To simplify the identification of the training and test sets derived from the original dataset, we call $T_{r}$ the training set and $T_{e}$ the test set.

#### 3.1.5. Superimposition

**Figure 11:**
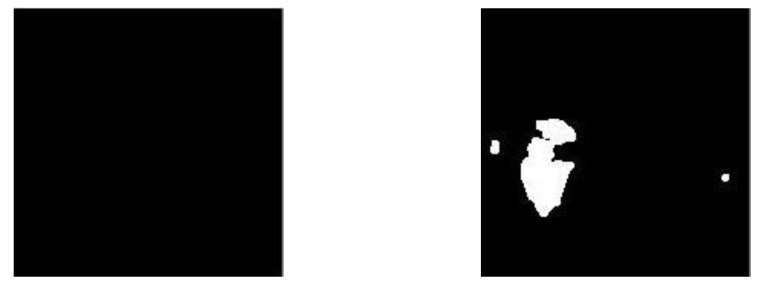
Result of the application of morphological operators erosion and dilatation on a normal class pattern and one of class IVH.

**Figure 12:**
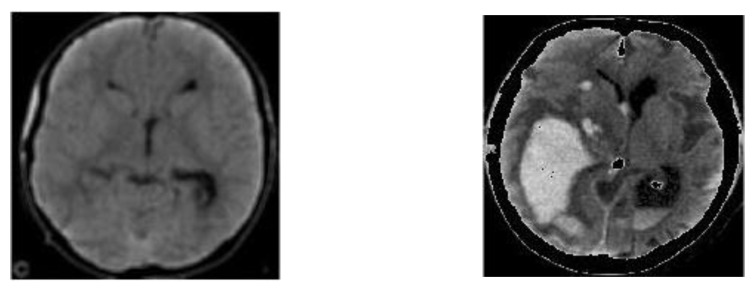
Result of the superimposition of images without bone tissue and areas with presence of hemorrhage.

In imaging, superimposition basically consists of superimposing an image over another existing image; usually, this technique is used to increase or de-emphasize features of the main or background image, but it can also be used to hide irrelevant information. In this work, once the segmented binary images were generated (see Figure 11), the segmented images with possible bleed-through, as shown in Figure 10, were superimposed over the equalized grayscale images, as shown in Figure 9. This creates an enhancement effect on the areas of interest [33] without losing the properties provided by the grayscale images provided by the dataset. Figure 12 shows the result of this superimposition.

This process is performed for the 252 images that make up the dataset but not before going through a flattening process to obtain patterns of 1×mn data where m is the height of each image and n is the width of each image.

### 3.2. Minimalist Machine Learning

In Section 3.2.1, Section 3.2.2 and Section 3.2.3, we will discuss in detail how the Learning and Testing phases are carried out, respectively, as well as the hypothesis function definition generated from the training process. In Section 3.3, we will show a couple of examples with real datasets, which will allow us to elucidate more precisely the process followed by the methodology proposed in this work. As mentioned in Section 2.4, this new paradigm bases its operation on the calculation of two basic mathematical operations: mean and standard deviation [17]. It is an iterative process that performs the search for the best hypothesis function that maximizes generalization and accuracy in the classification process in the supervised learning environment [37].

#### 3.2.1. Learning Phase

**Figure 13:**
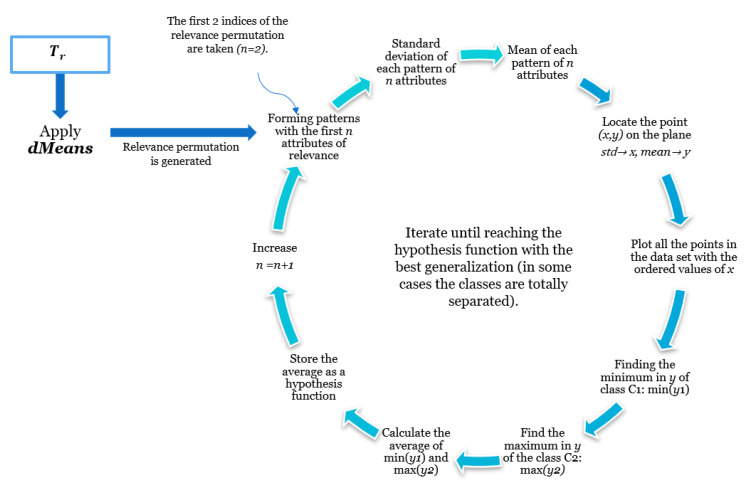
Algorithm of the training phase according to the MML paradigm.

The minimalist classifier learning phase algorithm is trained using the Minimalist Machine Learning paradigm, which consists of the iteration of a set of well-defined steps. These steps are described in Figure 13.

#### 3.2.2. Hypothesis Function

Part of the design of the classifier based on the new MML paradigm consists of defining a hypothesis function or decision boundary that allows the separation of two classes of a given dataset [38]. As shown in the previous figure (Figure 11), two fundamental measures are calculated for each of the patterns that make up the dataset: mean and standard deviation. By establishing both measures, it is possible to pose a function that, according to the definition of the MML paradigm, consists of defining a horizontal line that generalizes the separation of classes in the best possible way [17].

According to the MML paradigm, it is possible to obtain a horizontal line that partially or totally separates the classes, in order to generate a decision boundary that achieves a good performance in the classification of patterns [17]. It is important to mention that in some datasets, there is a separation between the two classes; the separation is total, reaching the maximum performance.

The hypothesis function or decision frontier is defined with a simple calculation, which consists of calculating the average distance between the maximum value referring to the lowest class and the minimum value referring to the highest class. This function is defined in the following equation:

(7) $$\mathrm{Hypothesis}=\frac{\max\left(minor\;Class\right)+\min\left(major\;Class\right)}{2}.$$

To assign a class label to a given test pattern p, the following function is applied:

(8) $$\mathrm{Assigned}\;\mathrm{Class}=\{\begin{array}{c} \mathrm{Class}\;\mathrm{One}\;\;\;\;\;\;if\;mean\left(p\right)<Hypothesis\\ \mathrm{Class}\;\mathrm{Two}\;\;\;\;\;\;if\;mean\left(p\right)>Hypothesis \end{array}.$$

Graphically, this horizontal line defined by the hypothesis function will allow distinguishing those test examples that are correctly or incorrectly classified.

#### 3.2.3. Test Phase

**Figure 14:**
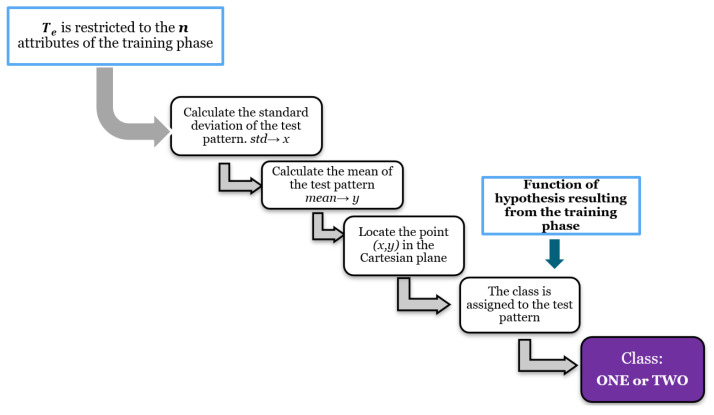
Algorithm of the classification phase according to the MML paradigm.

Executing the test phase basically consists of calculating the aforementioned measures: mean and standard deviation, and evaluating the position with respect to the hypothesis function resulting from the learning phase. The Leave-One-Out Cross-Validation method was implemented. The set $T_{e}$ consists of only one pattern, which was not considered in the training of the model [39]. Figure 14 illustrates the process related to the testing or classification phase.

### 3.3. Illustrative Examples of Minimalist Machine Learning

In order to know in more detail how the Minimalist Machine Learning paradigm works, and its capacity to be applied to a pattern classifier, we decided to show step by step how this novel paradigm works, applying it to a couple of examples with real datasets. The development of these illustrative examples aims to elucidate the doubts generated about the functioning of the MML paradigm. In addition, through this pair of examples, two different behaviors will be observed when applying the MML algorithm on two different Datasets.

#### Example 1: Gordon Dataset

A dataset was chosen that has the characteristic of behaving “very well” with the MML paradigm. It is the Gordon dataset [40]. The data in the dataset consist of data obtained from preprocessor lung cancer testing of 1627 genes from 181 lung cancer patients. Of the total, 31 of these patients have Adenocarcinoma (AD) and 150 patients have Malignant Pleural Mesothelioma (MPM).

Particularly, in this example, it will be possible to observe graphically how the MML paradigm works, in a dataset that achieves its total separation, that is to say, 100% is reached in the calculated performance measures.

**Figure 15:**
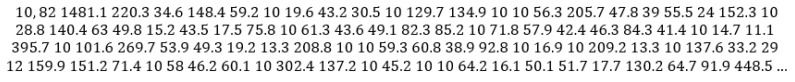
Section of the first MPM class pattern extracted from the original dataset to be part of the test set.

**Step 1:** According to the LOOCV validation method, the first pattern belonging to the MPM class is separated in such a way that it conforms the test set. Consequently, the remaining 180 patterns become the training set (this process will be iterated with each of the 181 patterns that make up the dataset). To exemplify, Figure 15 shows a section of the first test pattern.

**Figure 16:**
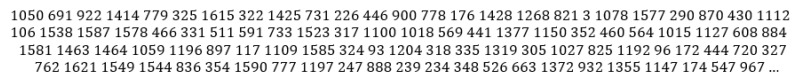
Segment of the attribute relevance permutation, resulting from the application of the *dMeans* algorithm.

**Step 2:** After having separated the first pattern of the MPM class to become the test set, the remaining 180 patterns form the training set. On this training set of 180 patterns, the *dMeans* transform is applied, from which an array is obtained that orders from highest to lowest relevance each of the 1627 attributes of the Gordon dataset. After applying the *dMeans* transform, the relevance permutation is as shown in Figure 16. This means that attribute 1050 is the most relevant; 691 is the second most relevant; the third most relevant is 922, and so on, until reaching attribute 463, which is the least relevant of all.

**Figure 17:**
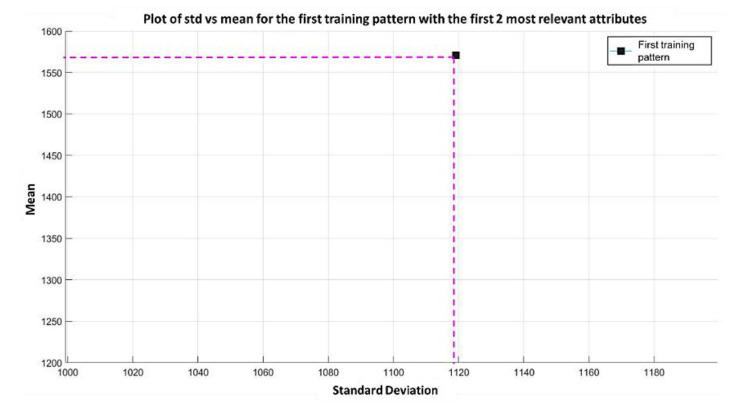
Representation of the first transformed training pattern.

**Step 3:** The next step in the learning phase of the MML paradigm consists of taking the two most relevant attributes and processing each of the patterns (these two attributes correspond to positions 1050 and 691, according to Figure 16). For example, in the first training pattern (which in this case is the second pattern of the MPM class) attribute 1050 has a value of 779.1, and attribute 691 has a value of 2361.8. The standard deviation of these two values is calculated, and it is placed on the x-axis; then, the mean is placed on the y-axis, and that allows locating a point on the Cartesian plane; see Figure 17.

**Figure 18:**
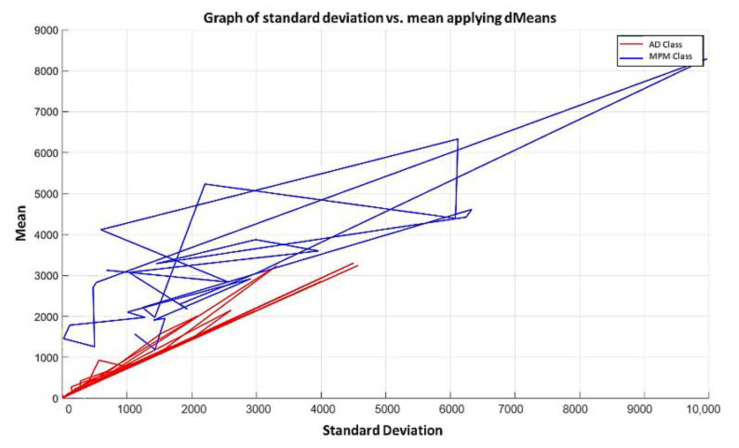
Training of the Gordon dataset, using the MML paradigm with two relevant attributes.

**Step 4:** We continue with the other training patterns until we reach the last instance of the dataset, which is the last pattern of the AD class. In this pattern, the attribute 1050 has a value of 67.9, and attribute 691 has a value of 81.6, so its mean is 74.75 and its standard deviation is equal to 9.6874. The standard deviation of each pair of values is calculated, and the resulting values are located on the x-axis. Then, the mean of that same pair of values is calculated, and the resulting mean values are located on the y-axis, corresponding to the values of the standard deviation of each pattern. The plot of the 180 training patterns considering only positions 1050 and 691 is shown in Figure 18.

**Figure 19:**
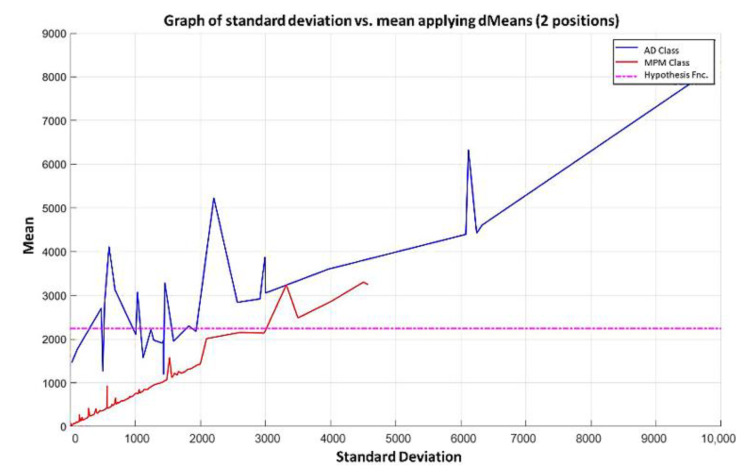
Modification of Figure 18 by ascending order of the standard deviation values on the x-axis. Standard deviation values on the x-axis.

**Step 5:** Following the algorithm of the MML paradigm, Figure 18 is transformed into Figure 19 where the values of the standard deviation have now been ordered, considering, of course, the values corresponding to the mean in each case.

NOTE: The magenta line in Figure 19 represents the hypothesis function that has been calculated. In the subsequent figures in the examples, the magenta line in each has a similar meaning.

Using the first two most relevant attributes according to the relevance permutation of Figure 16, it is noticeable that there is no line in Figure 19 that can separate the two classes. Therefore, following the algorithm of the MML paradigm, we must add one by one the values of the features of the positions specified by the relevance permutation of Figure 16.

**Figure 20:**
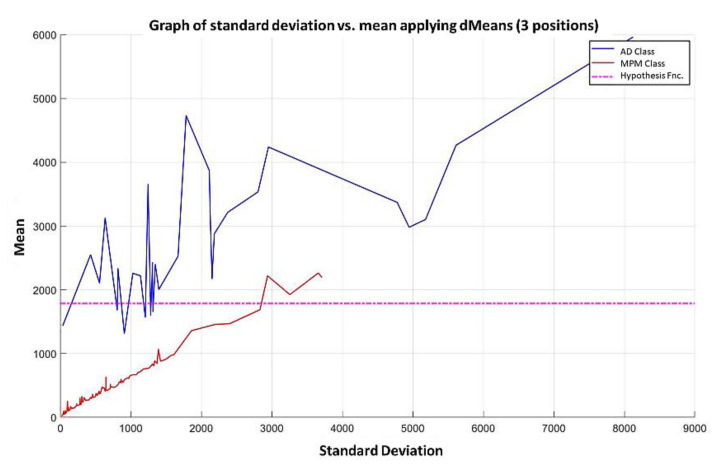
Training of the Gordon dataset using the MML paradigm with the three most relevant attributes.

**Step 6:** Let us see what happens when the first 3 most relevant attributes are used. Now, the 180 training patterns are restricted to the values indicated by the first 3 relevant attributes, which according to the relevance permutation in Figure 16 are the following: 1050, 691, and 922. The standard deviation for each learning pattern is calculated but now considering only the values corresponding to this triad and plotting on the x-axis the resulting standard deviation values. Then, the mean is calculated for each of the learning patterns considering the same triad, and the resulting mean values are placed on the y-axis, corresponding to the values of the standard deviation, as shown in Figure 20, where the ascending order according to the standard deviation has already been performed.

**Figure 21:**
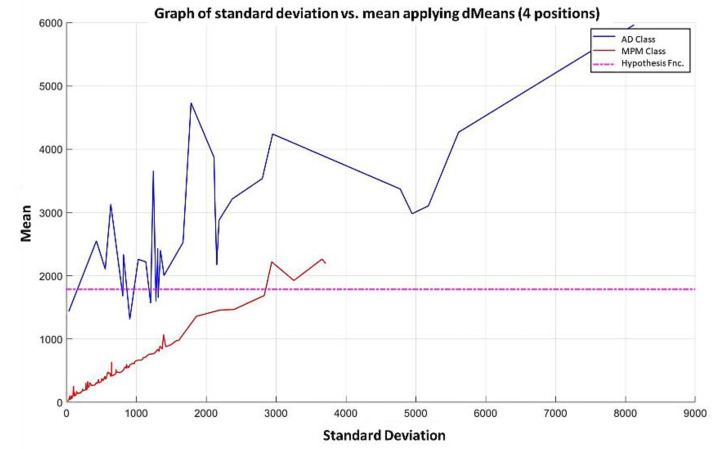
Training of the Gordon dataset using the MML paradigm with the four most relevant attributes.

**Figure 22:**
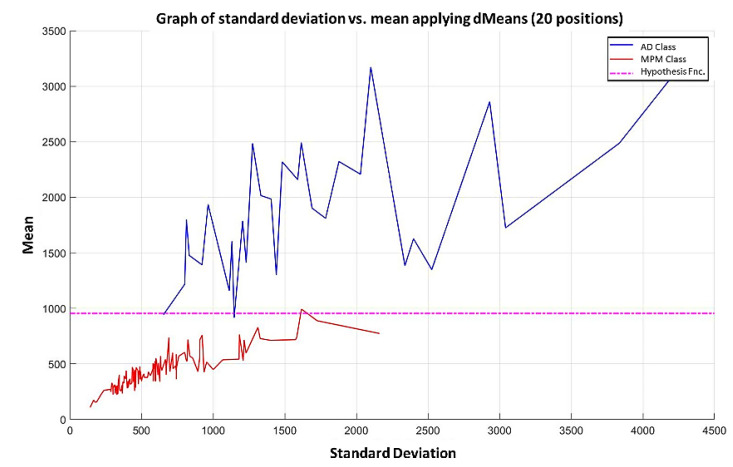
Training of the Gordon dataset using the MML paradigm with the 20 most relevant attributes.

**Figure 23:**
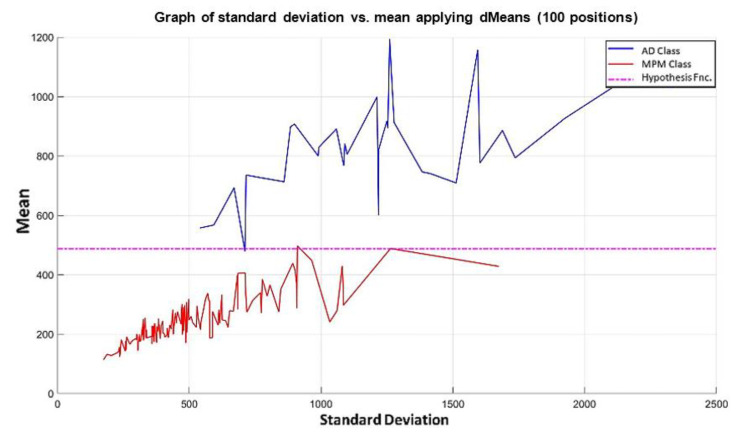
Training of the Gordon dataset using the MML paradigm with the 100 most relevant attributes.

**Figure 24:**
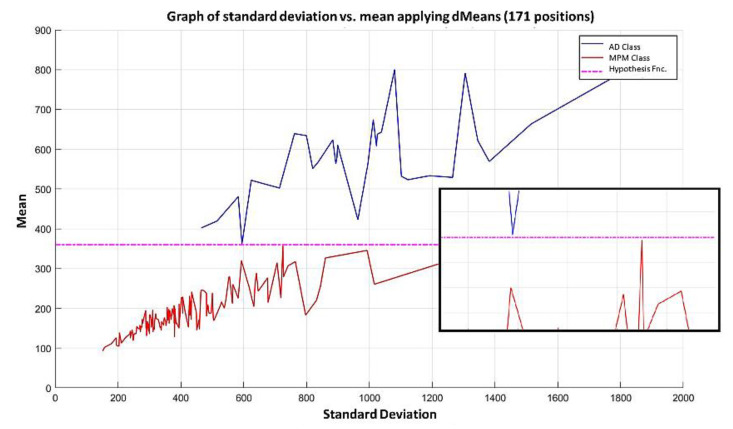
Training the Gordon dataset using the MML paradigm with the 171 most relevant attributes. This is achieved with the first 171 positions of the relevance permutation in Figure 23; this ends the learning phase.

**Step 7:** Subsequently, attributes continue to be added according to the re-relevance permutation of Figure 16 until the maximum separation of the classes is achieved. This behavior is illustrated in Figure 21, Figure 22, Figure 23 and Figure 24, where the number of relevant attributes has been increased to 171.

The training of the Gordon dataset using the MML paradigm concludes at the moment when each pattern is formed with the first 171 most relevant attributes according to the relevance permutation in Figure 16. This means that at the conclusion of this step, we have three data at the exit of the learning phase.

1. The positions of the first 171 most relevant attributes.
2. The line representing the hypothesis function, which in this case is the value 359.63 on the y-axis. This value was calculated using expression 3.3.
3. This third piece of data is very important for the classification phase: the AD class (blue) is above the hypothesis line, while the MPM class (red) is below the hypothesis line.

It is possible to observe that by increasing the number of relevant attributes in the training process, the separation of the classes becomes more and more evident; and through the application of the MML paradigm, it is observed that the total separation of the classes is achieved with the use of the first 171 attributes of greater relevance in Figure 16, as shown in Figure 24.

**Figure 25:**
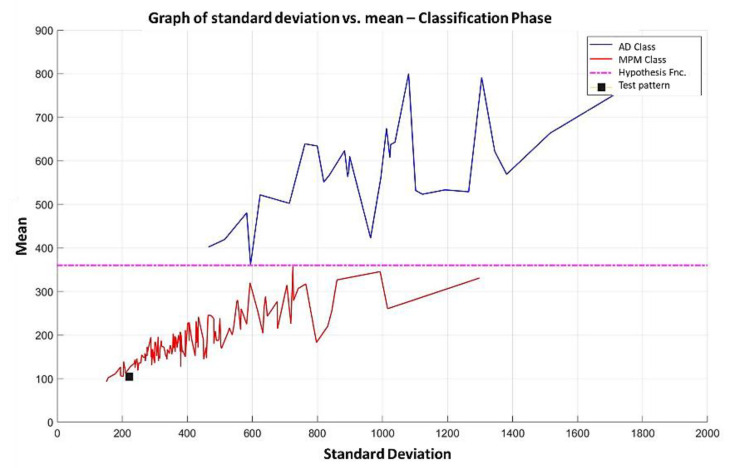
Representation of the classification phase of instance one in the Gordon dataset.

**Step 8:** After concluding the learning phase, we have the three data shown in the previous sections. With these 3 data, it is now possible for the MML paradigm to assign a class to the test pattern. To do so, the values of the 171 most relevant attributes are placed in Figure 24, which contains the 1627 attributes of the test pattern. With that test pattern restricted to the 171 positions specified in item 1 (which are the first 171 positions in Figure 24), the standard deviation and mean are calculated, and the point is located on the Cartesian plane, as shown in Figure 25. This point is represented by the black square; as this black square is below the hypothesis line, the conclusion is direct, and the test pattern should be assigned the MPM class (red). As the MML paradigm is supervised, it is possible to verify that this test pattern was correctly classified.

**Step 9:** By applying the procedure described in step 8, using each of the Gordon database standards in the cycle that corresponds to them as test standards, it is observed that all of them are correctly classified. This means that the values of the 3 performance measures are excellent: accuracy=1; sensibility=1; and specificity=1.

The results obtained in example 1 are excellent, and it is rarely found in the state of the art that a classifier, no matter how good it is, exhibits such good results in any database. The non-existence of the perfect classifier is guaranteed by the No Free Lunch Theorem [41].

As a consequence of the No Free Lunch theorem, it is important to point out that not all databases allow a total separation of their classes by means of the MML paradigm. Although it is possible to notice a greater difference between their classes, the separation of these is not 100% achieved, as was evident in the previous example. Therefore, an example case in which the classes are not completely separated will be shown below.

#### Example 2: Adenocarcinoma Dataset

In this example, the MML paradigm will be applied to the Adenocarcinoma dataset, which contains data from 76 patients; 64 of these have Primary Tumors (PT) and 12 have Metastatic Tumors (MT). For each of the patients, there are 16,063 genes or attributes [42]. For this example, the reduced version with 9868 attributes will be used [43].

**Figure 26:**
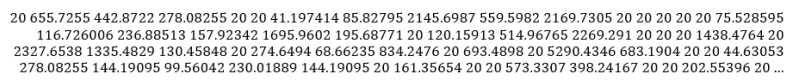
Fragment of the first pattern of the TP class, extracted from the original dataset, converted into a test pattern.

**Step 1:** The first pattern belonging to the TP class, now converted into the test set according to the LOOCV validation method, is separated. A fragment of the 9868 attributes is listed (Figure 26).

**Figure 27:**
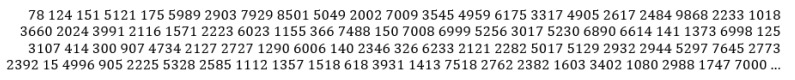
Fragment of attribute relevance permutation resulting from the *dMeans* algorithm.

**Step 2:** After having separated the first pattern of the TP class to become the test set, the remaining 75 patterns form the training set. On this training set of 75 patterns, the *dMeans* transform is applied, from which an array is obtained that orders each of the attributes of the Adenocarcinoma dataset in order of relevance. After applying the *dMeans* transform, the relevance permutation is as shown in Figure 27. This means that attribute 78 is the most relevant; 124 is the second most relevant; the third most relevant is 151; and so on until the least relevant of all.

**Figure 28:**
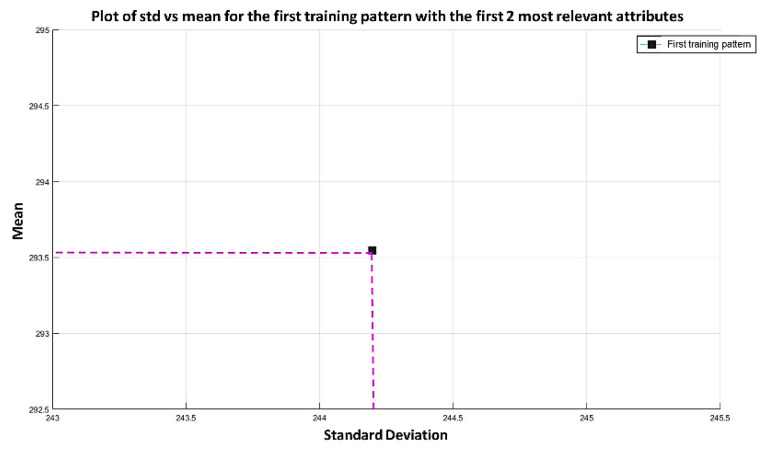
Representation of the first transformed training pattern.

**Step 3:** The next step in the learning part of the MML paradigm consists of taking the two most relevant attributes (78 and 124, according to Figure 27), extracting the values they contain, and plotting them on the Cartesian plane, following the same procedure as described in point 3 of Example 1, resulting in Figure 28.

**Figure 29:**
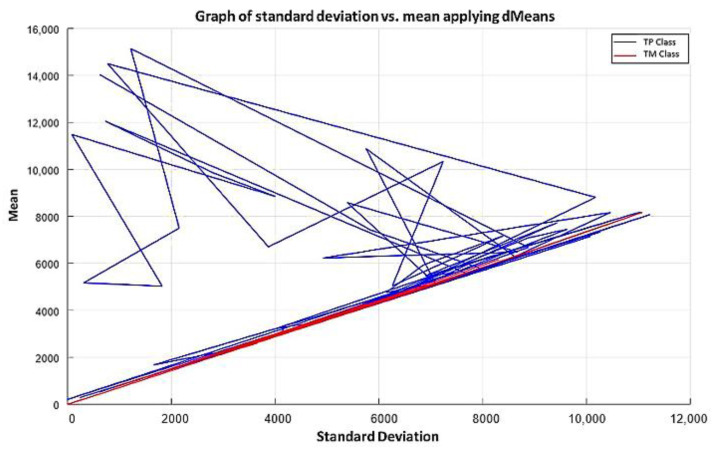
Training of the Adenocarcinoma dataset using the MML paradigm with two relevant attributes.

**Step 4:** Subsequently, we continue with the other training patterns until we reach the last instance of the TM class. The graph of the 75 training patterns is shown in Figure 29, analogously to step 4 of example 1.

**Figure 30:**
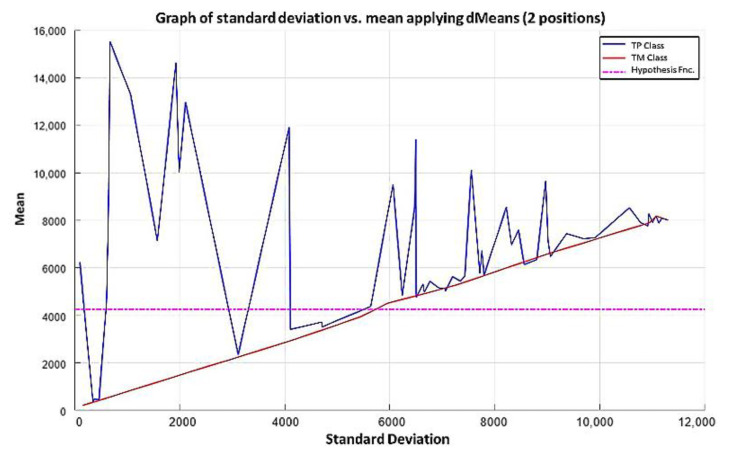
Training the Adenocarcinoma dataset using the MML paradigm with two relevant attributes.

**Step 5:** Continuing with the algorithm of the MML paradigm, Figure 29 is transformed into Figure 30 where the standard deviation values have now been ordered with their respective mean values.

**Figure 31:**
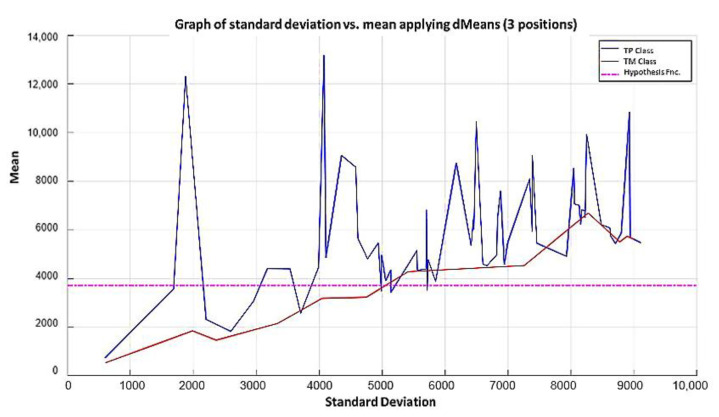
Training of the Adenocarcinoma dataset using the MML paradigm with three relevant attributes.

**Figure 32:**
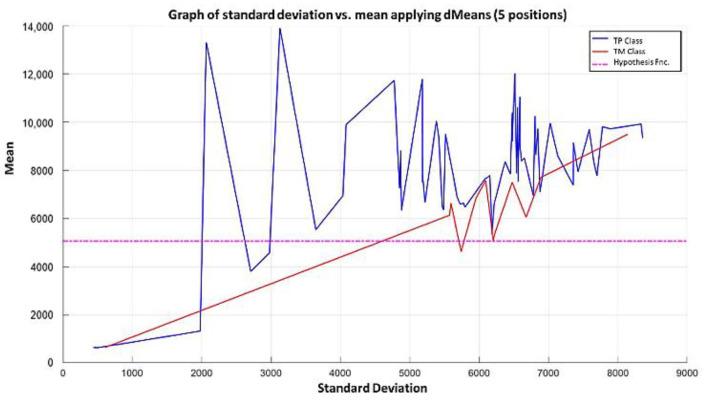
Training of the Adenocarcinoma dataset using the MML paradigm with five relevant attributes.

**Figure 33:**
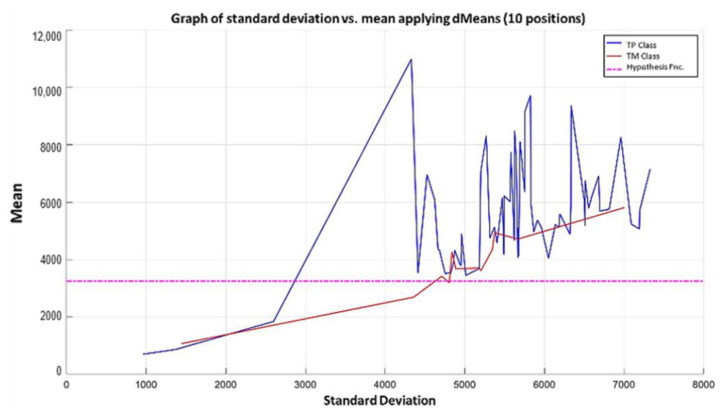
Training the Adenocarcinoma dataset using the MML paradigm with 10 relevant attributes.

**Figure 34:**
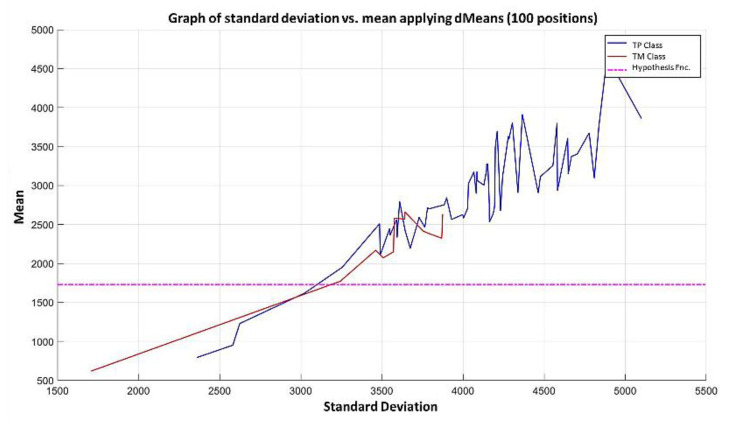
Training the Adenocarcinoma dataset using the MML paradigm with 100 relevant attributes.

**Figure 35:**
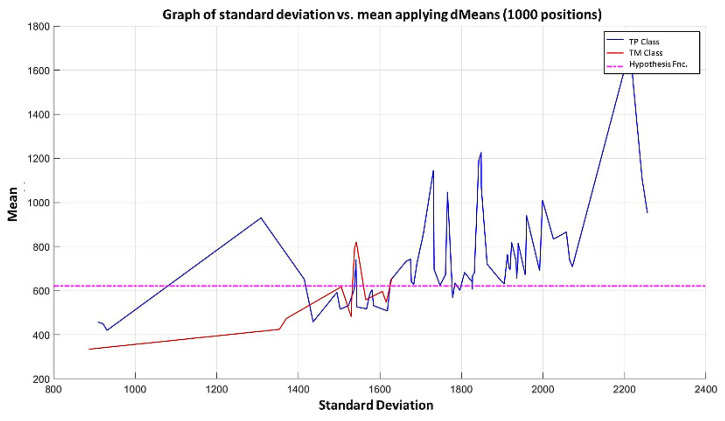
Training the Adenocarcinoma dataset using the MML paradigm with 1000 relevant attributes.

**Figure 36:**
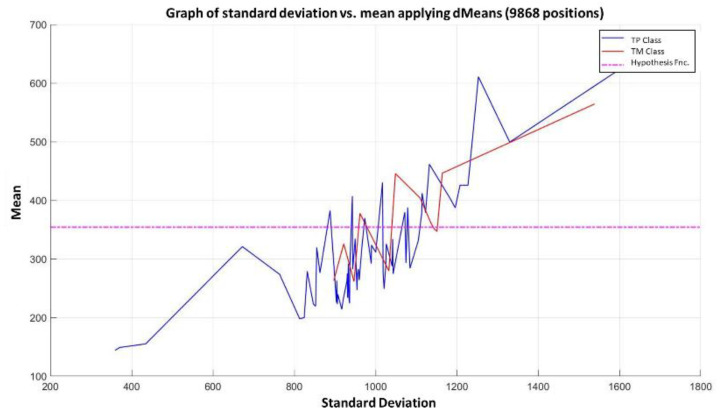
Training of the Adenocarcinoma dataset using the MML paradigm with all relevant attributes.

**Step 6:** To simplify the iterative process in increasing the use of attributes of higher relevance, the results generated by the MML paradigm will be shown for a different number of attributes of relevance, taking 3 (Figure 31), 5 (Figure 32), 10 (Figure 33), 100 (Figure 34), 1000 (Figure 35), and 9868 attributes (Figure 36), and showing the maximum class separation when taking the first 1000 attributes. However, as mentioned at the beginning of the description of this example, the total separation of the classes is not possible.

It is possible to observe that by increasing the number of relevant attributes in the training process, the separation between both classes becomes noticeable; however, this is one of the cases in which the MML paradigm does not achieve the total separation of the classes of the dataset, reaching its maximum separation with the use of the first 1000 attributes of greater relevance.

**Figure 37:**
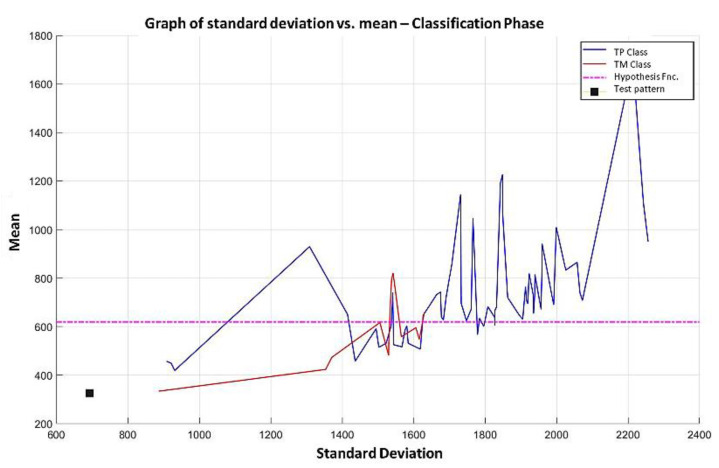
Representation of the classification phase of instance one in the Adenocarcinoma databank with an incorrectly classified test pattern.

**Figure 38:**
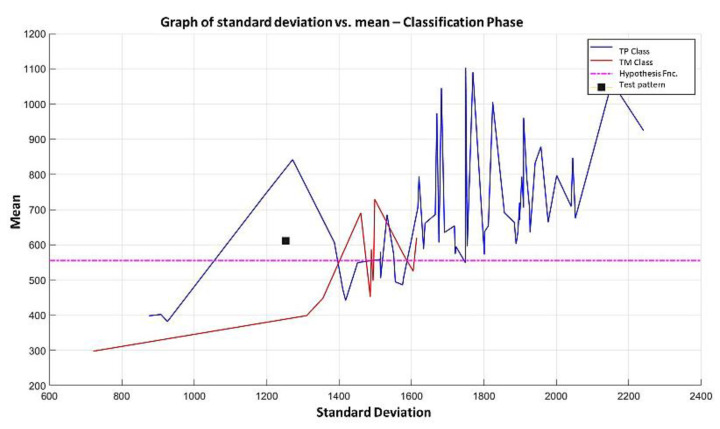
Representation of the classification phase of instance one in the Adenocarcinoma databank with a correctly classified test pattern.

**Step 7:** After concluding the learning phase, the 3 data shown in steps 1, 2 and 3 of point 7 of Example 1 are available. For this purpose, the values of the 1000 most relevant attributes are placed in Figure 27, which contains the 9868 attributes of the test pattern. That test pattern is restricted to the 1000 positions specified in item 1 (which are the first 1000 positions in Figure 35). The standard deviation and mean are calculated, and the point is located on the Cartesian plane, as shown in Figure 37, which shows the case of a test pattern classified incorrectly, and Figure 38 shows the case of a test pattern classified correctly. This point is represented by the black box; and as this black box is below the hypothesis line, the conclusion is straightforward, and the test pattern should be assigned the MPM class (red). As the MML paradigm is supervised, it is possible to verify that this test pattern was correctly classified.

**Step 8:** When applying the procedure described in step 7, in which each of the Adenocarcinoma databank standards in the cycle that corresponds to them are test standards, the following performance measures are observed: sensibility= 0.333; specificity= 0.8493 and accuracy= 0.8267.

The results obtained in example 2 are no longer as good as those in example 1, which is supported by the No Free Lunch Theorem [41].

## 4. Results and Discussion

This results section shows the experimental framework for the development of this work. A description of the dataset used to demonstrate the performance of this new paradigm in performing the CT image classification task and the performance comparison with other models reviewed in the state of the art is shown. In addition, comparative tables with different performance measures are presented to elucidate the results obtained as well as the time elapsed in each experiment.

### 4.1. Dataset

In this subsection, the dataset used for the experimentation and application of the novel Minimalist Machine Learning paradigm will be mentioned. This set is made up of images obtained by Computed Tomography (CT); therefore, they have the characteristics of being formed by positive integers and lack missing values.

#### DS: Brain Hemorrhage CT Dataset

The third dataset used in this paper was the Brain Hemorrhage CT image set [18]. To evaluate the performance of the proposed algorithm, an image bank of 627 images of five different classes (HED, SHD, SAH, IVH, and Normal) was used; originally, the dimensions of all images were 128 x 128 pixels in JPG format and in a grayscale representation. They are images acquired by the Computed Tomography method in people between 15 and 60 years of age. For this experiment, only one class was taken from those suffering from Intra-Ventricular Hemorrhage (IVH) and Non-Hemorrhage type, forming a final dataset of 252 images (129 of IVH class and 123 of Normal class) whose imbalance rate is approximately IR≅1.05 [36].

### 4.2. State of the Art Classifiers for Comparison

#### 4.2.1. K-Nearest Neighbors (K-NN)

The 1-NN (1-Nearest Neighbor) is a simple classifier, in which each pixel is classified into the class in which the training set presents the highest intensity. However, there is a possibility of misclassification if the single nearest neighbor is an outlier of some other class, and to avoid such errors and improve robustness, K-NN is intended to work with K instances [44]. Since it is practically a nearest-neighbor computation, it is considered a non-parametric model, since it does not consider any statistical property of the dataset [23]. The pseudocode that represents the operation of the KNN algorithm is shown in Algorithm 2.
**Algorithm 2:** K-NN Classification algorithm.**Input:**$T_{e}$ Training set, with defined classes C
1. Training set $T_{e}$ with classes C included.2. Examine the K elements close to the pattern to be classified.3. A new element is placed in the class with the highest number of nearby elements.4. The process is repeated for each tuple to be classified (depending on the size of the training set).**Output:** Class label for the test pattern.

The classification of K nearest neighbors is performed by a training set that contains the information about each input pattern and is compared with the test patterns. The distance of the unknown patterns to the K nearest neighbors determines the assignment of their class, either by majority vote or by averaging the values of the nearest classes [44].

#### 4.2.2. Multi-Layer Perceptron (MLP)

For medical diagnostic decision making, there is a great variety of artificial neural networks, and MLP networks are commonly used because of their simple architecture, their great capacity to be represented, and their easy access through not so sophisticated computing equipment and with a great number of programmable algorithms with different varieties of architectures [45].

Generally, an MLP consists of three layers: an input layer to the training examples, a hidden layer that will adjust the synaptic weights, and an output layer [46].

The training algorithm of the MLP model is described in the following pseudocode shown (Algorithm 3).
**Algorithm 3:** MLP Classification Algorithm.**Input:**$T_{e}$ training set, with classes desired outputs C.1. Choose a vector of initial weights w.2. Initialize the approximation to cost minimization.**while** the error does not converge **do****for** all patterns p
**do**
1. Apply the p patterns to the network and compute the network output.
2. Calculate ∂E.
3. For all weights, sum over all training patterns.
4. Update the weights in each error minimization approximation pattern.**end while****Output:** Approximate class label for the test pattern.

#### 4.2.3. Support Vector Machine

Support Vector Machines (SVMs) are powerful supervised classification systems with very accurate learning techniques that were introduced in 1995 [47]. SVMs have been shown to have higher efficiency than Artificial Neural Networks (ANNs) and Radial Basis Functions (RBFs), as SVMs use optimized linear separators; e.g., separation is possible by using hyperplanes to separate two datasets in an attribute space.

**Figure 39:**
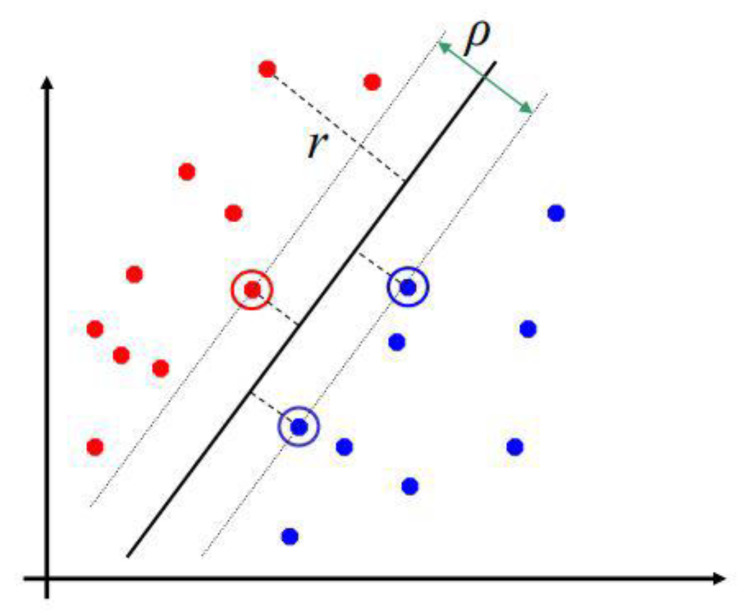
Representation of the maximization of the minimum class separation margin.

This optimization of the hyperplane is achieved by maximizing the minimum margin between the two sets (see Figure 39).

For the calculation of the distance of the training instance to the linear separator, the expression $r=\frac{w^{T}x+b}{\left|\left|w\right|\right|}$ is used; from this expression, the instances that give support to this hyperplane are derived, which are known as support vectors, and the margin is the margin of separation between both classes. Therefore, the hyperplane depends only on the boundary between the training patterns.

SVMs work under two mathematical operations:

- Non-linear representation of an input vector in a higher dimensional feature space.
- Construction of an optimal hyperplane to separate the attributes [47].

Data that are able to be linearly separated can be analyzed and classified by using a hyperplane; otherwise, if the data present a non-linear behavior, the data are classified by using kernel (K) functions, such as Linear SVM [48] and Gaussian RBF [49].

(9) $$K\left(x,y\right)=x^{T}y\;\mathrm{Linear}\;\mathrm{SVM}$$

(10) $$K\left(x,y\right)=\exp\left(-\frac{{\left|\left|x-y\right|\right|}^{2}}{\sigma^{2}\;}\right)\;\mathrm{Gaussian}\;\mathrm{RBF}$$

Related to image classification, the most commonly used kernel is RBF when it comes to classifying two-class datasets.

#### 4.2.4. Adaboost

Robert Schapire originally introduced boosting-based classification algorithms in the context of Machine Learning. These algorithms consist of combining a set of classifiers with low performance to create a high-performance algorithm [50]. Adaboost is a widely used implementation for two-class classification.

Basically, this classifier trains the model under several types of classification algorithms, and it observes the error that is generated for each of them; observing what is the performance of each one, it assigns a weight to each of the algorithms in between, so that it can generate the combination of weak algorithms, which boosts the performance in the new strong classifier. Mathematically, the Adaboost algorithm is described by the following equation [51]:

(11) $$H\left(x\right)=sign\left(\overset{T}{\underset{t=1}{\sum }}\alpha_{t}\cdot h_{t}\left(x\right)\right)$$

where T are the weak classifiers to be evaluated; $h_{T}\left(x\right)$ is the output of the weak classifier t, which is regularly limited to values −1 or +1. $\alpha_{t}$ is the weight applied to classifier t determined by Adaboost. Finally, the output is the linear combination of all weak classifiers evaluated.

#### 4.2.5. Random Forest

Random Forest decision trees are classification algorithms where their performance consists of the combination of numerous Decision Trees (DT), which are named after their tree-like structure. Each DT of the RF depends on the values of a random vector sampled independently and with the same distribution. After a certain number of trees have been generated, all input variables and all possible splitting points are evaluated and the most popular class is voted on; this procedure is known as Random Forest [52].

RF is a classifier consisting of a collection of classifiers with tree structure {h(x, k), k=1, …} where the {k} values are independently distributed random vectors and each tree casts a unit vote for the most popular class on the x input. To implement RF, it is necessary to set the number of iterations or number of trees $n_{tree}$, and the number of features in each split m. Several studies have shown that satisfactory results can be found with default parameters. Several studies, such as the one performed by Liaw and Wiener [53], describe that a high number of trees gives a more stable result compared to trees with a lower number of iterations.

#### 4.2.6. Naïve Bayes

Naïve Bayes (NB) is a simple classification algorithm that is purely probabilistic in origin. The result obtained by the probabilistic classifier is given by $P_{r}(C|d)$, where d is the probability that a pattern belongs to a class C, since each of the patterns contains features to which probabilities are assigned based on the number of occurrences within a particular dataset

Basically, the Naïve Bayes classifier consists of making an assumption about the values of the features, and these are conditionally independent of their target value [54]. This assumption states that given the target value of a given pattern, the probability of observing the conjunction $c_{1},\;c_{2},\;\ldots ,\;c_{n}$, is the product of the probabilities of each attribute.

(12) $$P\left(a_{1},\;a_{2},\;\ldots ,\;a_{n}|v_{j}\right)=argmax_{v_{j}\in V}\underset{i}{\prod }P(c_{i}|v_{j})$$

In this way, the NB classifier ignores possible dependencies between features.

#### 4.2.7. Hierarchical Classifier

This classifier, proposed by Shahangian et al. [33], generates an automatic algorithm for image segmentation and classification for IVH detection. Using a modified version of Distance Regularized Level Set Evolution, they separate the areas with the presence of hemorrhage. Then, they extract a defined set of features from the hemorrhages and generate a synthetic feature that takes into account as extra information from each hemorrhage. The authors then apply Attribute Selection techniques. Finally, using a Hierarchical classification structure, the Normal and IVH classes are classified.

All algorithms were ran using the Machine Learning auxiliary platform WEKA with the exception of the proposal of this work, which was developed in MATLAB code. A personal computer with a Windows 10 operating system was used, with an Intel (R) Core (TM) i7 CPU 8750H processor at 2.20 HGz, with 32 GB of RAM. Table 1 shows the parameters used for each classification algorithm implemented in the WEKA platform.

### 4.3. Performance and Comparative Analysis

The objective of any classification model is to predict a possible response based on previously labeled observations, and when tested with new observations or instances that have never been shown to the model before, this model generates a class assignment response for that new instance shown. In order to obtain more accurate approximations, different validation methods are used to divide the fundamental set; this division generates two subsets previously called training and test (which can also be found as validation). The training set will allow training the classification model, and the second set is used to test that the hypothesis generated from the training works correctly for instances not shown during training or learning [20,55].

The idea of these validation methods is mainly that the model must fit a perfectly defined training set with its labels and be evaluated with the remaining set (from them to evaluate a metric that allows knowing its performance). Before knowing the method chosen to know its performance, some basic concepts are defined.

- Training set: data that are shown to the model to be trained.
- Test set: data that the model has never observed and under which the performance of the classifier will be obtained.

#### 4.3.1. Leave-One-Out Cross-Validation (LOOCV)

The LOOCV method consists of iterating the training process of the fundamental set of observations except one, given that the fundamental set consists of m instances, the model will be trained with m−1, and the remaining one will have the function of acting as a test example. This process is repeated as many times as the number of instances contained in the dataset, serially excluding one element of these examples to take the place of the test example [55].

This validation method has great advantages over other methods with randomized permutations, since it substantially reduces the variance caused by segmenting the dataset into two parts and always considering the totality of the examples; in addition, the LOOCV results are easily and perfectly repeatable [55].

**Figure 40:**
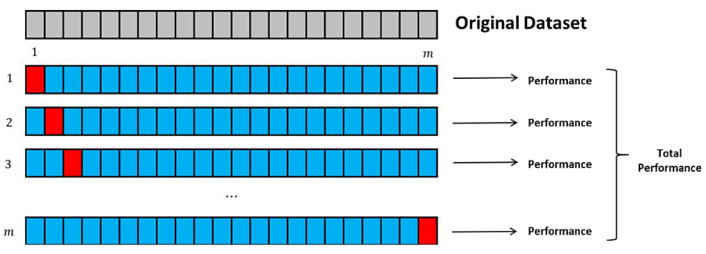
Example of LOOCV method behavior. In red color, the test example is shown. In blue color, the training examples.

Finally, as shown in Figure 40, each iteration generates a performance considering the number of hits and errors to finally be averaged and obtain a final performance.

#### 4.3.2. Performance Metrics

**Figure 41:**
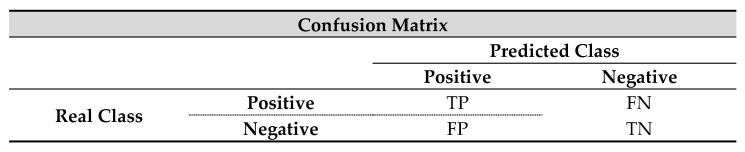
Confusion matrix for a two-class problem.

To generate results of the behavior of a model, it is essential to have a metric that allows knowing and comparing how good the result or the performance of a model is [56]. For this purpose, there are a large number of metrics, and each of them allows observing specific behaviors of the system. For this purpose, a matrix is used that allows appreciating the type of error or success that was generated, depending on the type of error or success that occurred. In this work, a two-class confusion matrix is used: positive class and negative class. For each of the four cells are designated certain values known mainly as True Positive (TP), True Negative (TN), False Positive (FP), and False Negative (FN). These are ordered as follows (Figure 41) [57].

Here:

- TP is the number of correct classifications of a positive pattern.
- TN is the number of correct classifications of a negative pattern.
- FP is the number of incorrect classifications of a positive pattern.
- FN is the number of incorrect classifications of a negative pattern.

The performance measure that will allow establishing a reference of the quality of the model will be the accuracy, since under this condition, it can be evaluated with full reliability of the model [58]. This measure is calculated by means of the following equation:

(13) $$Accuracy=\frac{TP+TN}{TP+TN+FP+FN}.$$

In addition to this, the measures Specificity and Sensitivity will also be calculated, as it is important to recognize the reliability of the model, depending on the type of misclassifications that occur [59]. They are widely used and robust under unbalanced datasets and can be easily obtained from a confusion matrix. The calculation of these measures is dictated as expressed in the following equations.

(14) $$Sensitivity=\frac{TP}{TP+FN}$$

(15) $$Specificity=\frac{TN}{TN+FP}$$

In the pattern classification task, Accuracy takes into account the number of correctly labeled patterns in relation to the total number of test patterns that underwent the classification phase. Sensitivity corresponds to the ratio of predicted positive cases to actual positive cases; the number of false negatives should be as close to zero as possible to generate reliability in the results. This would indicate that there are no errors in the diagnosis or positive prediction of a possible disease. Specificity refers to the proportion of negative prognoses, determining how many of the negative predictions were actually negative, taking into account the number of false positives predicted.

Having given the performance metrics used, the results achieved by the MLM paradigm classifier are shown below, in comparison with seven of the state-of-the-art classifiers. The comparative analysis is performed using the measures previously described in this subsection. In turn, a brief significance analysis is carried out to determine the confidence level of the results observed.

Table 2 shows the results obtained from the classifiers on the BHCT dataset, in the three different performance measures proposed: Accuracy, Sensitivity, and Specificity. Training time was also added as a measure of comparison of our proposal.

**Figure 42:**
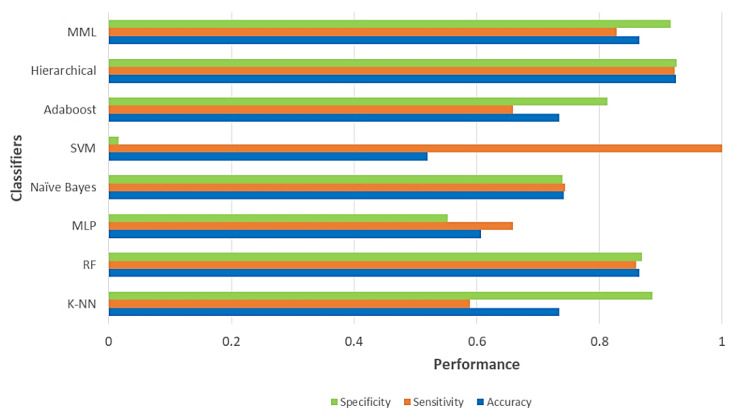
Comparison of performances with the state-of-the-art methods.

Figure 42 graphically illustrates the comparison between the classification algorithms tested, and it is clearly visible how competitive our proposal is with algorithms of great scientific relevance. It manages to maintain stability in its three performance measures, remaining extremely close to the best algorithm reported, and with the great advantage of remaining a simple, straightforward, and easily understandable algorithm: a characteristic that is not easy to find in most of the state-of-the-art methods.

As previously mentioned in Section 3.2, our proposal seeks to generate a solution that, in addition to showing competitive results in the different performance measures, allows understanding and representing in a simple way the training and classification process of any dataset. Moreover, through the application of the *dMeans* algorithm, and the learning process of the MML algorithm, additional results were obtained.

**Figure 43:**
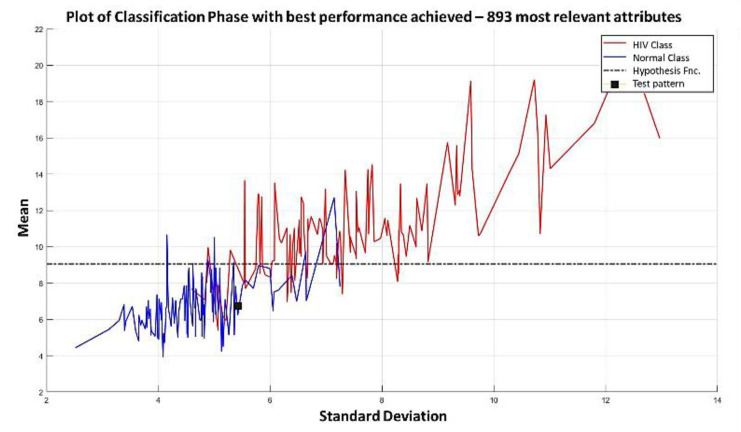
Final plot of the training and testing result of the MML paradigm (blue line: normal class; red line: IVH class; flashing line: hypothesis; rhombus: test example).

**Figure 44:**
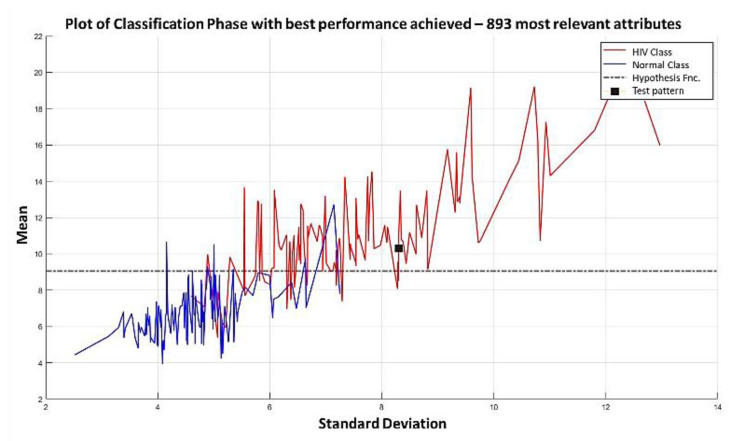
Final plot of the training and testing result of the MML paradigm (blue line: normal class; red line: IVH class; flashing line: hypothesis; rhombus: test example).

The relevance permutation obtained through *dMeans*, and the training process reaches its maximum performance with the first 893 most relevant attributes (Figure 43 and Figure 44).

It is evident that the training times are diverse for each of the classification models tested, with the K-NN classifier showing a wide advantage in terms of model training time. In general, most of the algorithms take between 100 and 200 s, with the two best models, RF and MML, showing very similar training times. However, the Hierarchical ranking algorithm is considerably faster, with only 60.57s of training time.

Regarding the results of the classification phase, it is evident that the MLP and SVM models present poor performances; meanwhile, the RF-based classifier has a homogeneous performance in its three performance measures, being the direct competitor against our proposal. Undoubtedly, the Hierarchical classifier maintains an above-average performance; this is understandable, as it is an ensemble of classifiers based mainly on SVM and Adaboost, besides applying Feature Selection techniques. The proposed MML algorithm has a competitive performance, reaching the second highest Accuracy value (0.8650), considering that, being a balanced dataset, it is a reliable and high-precision result, being surpassed only by the Hierarchical classifier that reaches an Accuracy value of 0.9262, which was only 6.12% better than the proposal itself. However, it is important to consider that the training time is among the highest shown in the state of the art.

Comparing the different Machine Learning algorithms, such as the classifiers shown in the state of the art and our main proposal, is of vital importance, as the evaluations or measurements calculated by means of cross-validation do not provide sufficient information to know the real abilities of each algorithm compared and thus rule out that the results shown are simply a statistical coincidence. In this sense, it was proposed to use Friedman’s test [60] to perform this comparison and find the statistical differences in the performances observed during the experimentation.

Table 3 shows the performances of the different classification algorithms proposed. These results show similar values. After performing Friedman’s statistical test, the null hypothesis was rejected with a confidence of 90% and a p-value of 0.0959, demonstrating the existence of statistically significant differences between the classifiers. Furthermore, the proposed MML methodology ranked second best in Friedman’s mean ranks measure for the comparative methods, while the MLP, Adaboost, and SVM algorithms remained with the highest averages, as shown in Table 3.

Finally, undoubtedly, the performance achieved by our main proposal could benefit from the application of much more sophisticated image segmentation and analysis techniques, as the results shown in Table 2 and Table 3 show the great potential of the MML paradigm. By implementing techniques based on multi-sequence Magnetic Resonance Imaging, more and better relevant information could be extracted from each of the images. These sequences could be such as, T1—weighted MRI (T1) [61], T1—weighted MRI with contrast enhancement (T1c) [61], T2—weighted MRI [61], Perfusion Weighted Imaging (PWI) [62], Diffusion Tensor Imaging [62], among others.

Each of these techniques has a specific purpose, which is determined by the type of element to be observed; similar to what has been done by F. Raschke et al. in the mapping of Glioma tissue types [62]. Specifically, in the detection of IVH, a set of Tissue Probability Mappings could be generated, each of these mappings ranging from White Matter, Gray Matter, and the four existing grades of IVH. Finally, recombining these mappings would allow generating a single image conformed by a superpixel mapping but with an important feature to be able to apply it to the MML paradigm, and that is that each superpixel mapping will not be conformed by segments in the RGB space but under a defined gray intensity for each of the IVH degrees found.

## 5. Conclusions and Future Works

In this paper, we have introduced a new classification algorithm of the Minimalistic Machine Learning paradigm. The classification algorithm based on the MML paradigm was implemented on a set of brain images obtained by Computed Tomography. Derived from the experimental work, a series of enhancement techniques were applied to this set of images for the improvement and enhancement of the areas of interest, specifically the areas with possible hemorrhage. As a result of our methodology, it has been shown to be highly effective in classifying between people who present Intra-Ventricular Hemorrhages and people without hemorrhages. Observing that the proposed method is competitive with other classification algorithms as K-NN, MLP, Naïve Bayes, SVM, Adaboost, and Hierarchical algorithms, ranking second on two performance measures and second only to the Hierarchical classifier on these two measures applied to the same dataset. It was also observed that the training time was less than that the SVMs and Adaboost. The substantial difference of our proposal lies in the ease in which the behavior of the algorithm and its results can be observed through a two-dimensional representation in the Cartesian plane and the minimization of necessary features, through the application of the *dMeans* method. This substantial improvement could allow the application of this methodology for mobile devices or embedded systems.

As well as the important advances achieved, it is important to mention the main limitations that come with the application of this new methodology for image classification. As could be evidenced in the results section, the training time is considerably high, considering the low number of images. Therefore, when applied in conjunction with a larger number of images and higher resolution, it would generate an extremely high computational cost, which is objectively the weakest point of the application of this model. For this reason, constant development is being carried out to reduce this effect in the Minimalist Machine Learning methodology. However, the excellent news lies in the generation of a classification model that meets all the indispensable characteristics of an algorithm belonging to the XAI, being extremely easy to explain, the interpretation of results is even graphical, which helps greatly to its interpretability, it is completely transparent, justifiable, and contestable to the sample of the results of their decisions.

As for future work, we plan to generate new algorithms under this novel Minimalistic Machine Learning paradigm and to continue experimenting with more datasets from different domains. In addition, considering the main limitations of this methodology, hard work will be maintained to substantially decrease the training time on similar datasets and of course seek to improve on the results seen during the development of this paper. As previously discussed at the end of the results section, the results shown can most likely be improved by applying advanced image segmentation techniques and applied to datasets of different characteristics, such as multi-sequence MR images. In addition, we plan to generate new pattern classification algorithms, applying Genetic Programming algorithms.

## Figures and Tables

**Table 1:** Parameter definition table used for the implemented classification algorithms.

Algorithm	Parameters
K-NN	K = 1; Distance Function = Euclidean Distance; Batch size = 100.
RF	Iterations = 100; Batch size = 100; Features = $\log_{2}\left(predictors\right)+1$; Depth = Unlimited.
MLP	Batch size = 100; Hidden Layers = 2; Learning rate = 0.3; momentum = 0.2; Epochs = 500.
Naïve Bayes	Batch size = 100.
SVM	Kernel = RBF; Batch size = 100; Kernel degree = 3; $\gamma =1/\underset{}{\max}\left(index\right)$.
Adaboost	Batch size = 100; Classifier = Decision Stump; Iterations = 10.
Hierarchical	Defined by the authors in [33].

**Table 2:** Performance of the compared classifiers in BHCT Images Dataset. ^1^ In seconds.

Algorithm	Accuracy	Sensitivity	Specificity	Training Time ^1^
K-NN	0.7341	0.5890	0.8862	6.07
RF	0.8650	0.8600	0.8700	203.11
MLP	0.6071	0.6590	0.5528	100.73
Naïve Bayes	0.7420	0.7440	0.7400	115.77
SVM	0.5198	**1.0000**	0.0162	752.23
Adaboost	0.7341	0.6590	0.8130	623.45
Hierarchical	**0.9246**	0.9231	**0.9262**	60.57
MML	0.8650	0.8280	0.9160	252.04

**Table 3:** Friedman mean ranks. ^2^ Ordered from best to worst.

Algorithm	Mean Ranks ^2^
Hierarchical	2.34
MML	3.83
RF	4.16
Naïve Bayes	6.00
K-NN	6.50
SVM	6.66
Adaboost	6.66
MLP	7.83

## Data Availability

Not applicable.
